# Can adverse childhood experiences predict chronic health conditions? Development of trauma-informed, explainable machine learning models

**DOI:** 10.3389/fpubh.2023.1309490

**Published:** 2024-01-15

**Authors:** Hanin B. Afzal, Tasfia Jahangir, Yiyang Mei, Annabelle Madden, Abeed Sarker, Sangmi Kim

**Affiliations:** ^1^Department of Mechanical and Industrial Engineering, University of Toronto, Toronto, ON, Canada; ^2^Department of Behavioral, Social and Health Education Sciences, Rollins School of Public Health, Emory University, Atlanta, GA, United States; ^3^School of Law, Emory University, Atlanta, GA, United States; ^4^Teachers College, Columbia University, New York, NY, United States; ^5^Department of Biomedical Informatics, School of Medicine, Emory University, Atlanta, GA, United States; ^6^Nell Hodgson Woodruff School of Nursing, Emory University, Atlanta, GA, United States

**Keywords:** behavioral risk factor surveillance survey, machine learning, adverse childhood experiences, chronic diseases, health behaviors, health outcomes

## Abstract

**Introduction:**

Decades of research have established the association between adverse childhood experiences (ACEs) and adult onset of chronic diseases, influenced by health behaviors and social determinants of health (SDoH). Machine Learning (ML) is a powerful tool for computing these complex associations and accurately predicting chronic health conditions.

**Methods:**

Using the 2021 Behavioral Risk Factor Surveillance Survey, we developed several ML models—random forest, logistic regression, support vector machine, Naïve Bayes, and K-Nearest Neighbor—over data from a sample of 52,268 respondents. We predicted 13 chronic health conditions based on ACE history, health behaviors, SDoH, and demographics. We further assessed each variable’s importance in outcome prediction for model interpretability. We evaluated model performance via the Area Under the Curve (AUC) score.

**Results:**

With the inclusion of data on ACEs, our models outperformed or demonstrated similar accuracies to existing models in the literature that used SDoH to predict health outcomes. The most accurate models predicted diabetes, pulmonary diseases, and heart attacks. The random forest model was the most effective for diabetes (AUC = 0.784) and heart attacks (AUC = 0.732), and the logistic regression model most accurately predicted pulmonary diseases (AUC = 0.753). The strongest predictors across models were age, ever monitored blood sugar or blood pressure, count of the monitoring behaviors for blood sugar or blood pressure, BMI, time of last cholesterol check, employment status, income, count of vaccines received, health insurance status, and total ACEs. A cumulative measure of ACEs was a stronger predictor than individual ACEs.

**Discussion:**

Our models can provide an interpretable, trauma-informed framework to identify and intervene with at-risk individuals early to prevent chronic health conditions and address their inequalities in the U.S.

## 1 Introduction

Adverse childhood experiences (ACEs) represent a critical public health issue. Defined as potentially traumatic events that occur in childhood (0–17 years old), ACEs include but are not limited to children experiencing emotional, physical, and sexual abuse, parental neglect, household instability such as parents’ divorce or separation, and suicide attempts (1). According to the Centers for Disease Control and Prevention (CDC), approximately 61% of adults surveyed across 25 states reported having experienced at least one ACE before adulthood; one in six claimed that they had experienced four or more ACEs (1). Despite the widespread prevalence of ACEs, some groups are at a higher risk of ACE exposure than others. For example, Black, Hispanic, or low-income individuals show the highest prevalence of ACEs (2). Additionally, social, economic, and environmental inequities are greater in the environments of those who have endured four or more ACEs (3).

Current literature has documented that experiences of maltreatment and psychosocial stress during childhood play a significant role in shaping a wide range of chronic health conditions, which constitute physical and mental health problems that last for a prolonged period (i.e., 1 year or longer) (4). The seminal ACE Study with 17,000 adults found a clear and strong correlation between the number of negative experiences during childhood and a wide spectrum of poor health and behavioral outcomes in adult life (5). The study demonstrates a dose–response relationship between the number of ACEs and chronic diseases (e.g., ischemic heart disease, cancer, and chronic lung disease) (5). Since then, mounting evidence indicates the positive associations between ACEs and chronic health conditions, including arthritis, pulmonary disease, cancers, cardiovascular disease, stroke, pre-diabetes, diabetes, high cholesterol, and renal disease (6–28). In addition, individuals with ACEs are found to be at greater risk of experiencing poor mental health (e.g., depression, anxiety, and hallucination) (29–36).

Multiple pathways connect ACEs to chronic health conditions, including social determinants of health (SDoH) and health behaviors. Individuals with a higher number of ACEs tend to live in areas of greater poverty, fewer economic and health resources, worse food access, less green space, and more community instability (3, 37, 38). ACE survivors are also more likely to engage in harmful behaviors, such as smoking, heavy alcohol consumption, substance use, high-risk sexual behavior, interpersonal violence, excess screen time, and inadequate sleep (5, 27, 30, 39–42).

Such clustering of social and disease conditions in a specific population is well-explained by syndemic theory. A syndemic is defined as the “aggregation of two or more diseases or other health conditions in a population in which there is some level of deleterious biological or behavior interface that exacerbates the negative health effects of any or all of the diseases involved” (43). In syndemics, social conditions contribute to disease formation, accumulation, spread, and progression by increasing susceptibility and reducing immune function, particularly among marginalized populations; hence, syndemics are most likely to emerge under conditions of health inequality (43). A syndemic can be exemplified by the interactions of ACEs, negative social conditions (i.e., SDoH), and risky health behaviors worsening the risk of various chronic health conditions (2, 3, 44). However, an accurate assessment of these complex associations can be methodologically challenging, as the involved risk factors may be highly correlated, interactive, or synergistic. In such cases, it is essential to employ models that are more flexible than linear regression, and robust at handling and computing features linked in nonlinear fashions. This need can be met by using more advanced modeling techniques such as machine learning (ML).

Most applications of Artificial Intelligence (AI) in healthcare read in categorical, numerical, or image-based data as an input; utilize algorithmic and statistical models to process the data; identify patterns; and produce a probability or classification (45–49). ML refers to the range of algorithms conducting these predictions (50). As briefly stated above, ML offers considerable benefits compared to traditional statistical modeling, as it is capable of handling complex multi-dimensional data, adapting new data as it becomes available, capturing non-linear relationships and interactions among variables more effectively, and generally accounting for noise and outliers in the data in a robust manner (50, 51). Moreover, ML can promote the P4 medicine paradigm—predictive, preventive, personalized, and participatory—an approach that proactively engages both providers and patients in early monitoring and intervention (52–54). For these reasons, there has been an exponential increase in using ML to predict the prognosis and outcome of chronic diseases.

Despite their advantages, however, health-related AI models are often impermeable black boxes: their inner workings are opaque, unintuitive, and uninterpretable to end-users. A lack of interpretability can compromise the end users’ trust and confidence in model predictions, especially when the model and its outcomes influence people’s decisions on their health and healthcare. In response to this growing need for transparency, explicability, and interpretability in AI models, the explainable AI (XAI) has emerged as a field. Today, XAI principles are applied for multiple purposes (e.g., reducing model bias toward certain racial or gender groups), and involve providing contextual information about the importance of variables in model decision-making (55).

Several existing studies have employed ML to predict an extensive range of chronic health conditions, such as autoimmune, cardiovascular, cerebrovascular, hepatic, metabolic, neurodegenerative, pulmonary, renal, and rheumatic diseases, as well as cancers (56–61). Most of these studies used K-nearest neighbors (KNN), support vector machines (SVM), Naïve Bayes (NB), deep neural networks, random forest (RF), and logistic regression (LR) (58, 60, 62–64). Existing classical ML models in the literature have predicted health outcomes based on SDoH with accuracies between 61 and 74% (65). It is common to combine different types and sources of data for these analyses, such as electronic medical records linked to omics data (63); clinical information linked to sociodemographic, behavioral, or anthropometric factors (58); and primary care data linked to insurance claims, cancer registries, or administrative sources (64). In terms of predictors, sociodemographic (e.g., age, sex, gender) and lifestyle factors (e.g., physical activity, lack of sleep, and use of alcohol, tobacco, and other drugs) are predominantly used for modeling chronic health conditions (58). However, only a small number of studies include ACE exposure in ML models to predict rheumatic and musculoskeletal disease (66), neurocognitive outcomes (67), and emergency department visits (68). Although a study by Ammar and Shaban-Nejad (69) proposes a proof-of-concept semantic XAI model for using ACEs and SDoH data to improve mental health surveillance, the model’s accuracy and usability are yet to be evaluated. Beyond these studies, few examine the use of ACEs in tandem with SDoH and health behaviors to predict a suite of chronic health conditions. Further, none of the previous studies use large national survey data to better represent the U.S. adult population.

The current study attempts to fill these gaps by developing interpretable ML models aimed at (i) predicting 13 chronic health conditions based on demographic characteristics, ACEs, SDoH, and health behaviors among U.S. adults and (ii) explaining the relative importance of variables in predicting each of the chronic health conditions. We use data from the CDC’s Behavioral Risk Factor Surveillance System (BRFSS), the world’s largest continuing national health survey (70). We employ classical ML models identified in the literature as robust tools for predicting chronic health conditions: LR, Gaussian NB, SVM, RF, and KNN (58, 60, 62–64) Although neural networks are also promising for this prediction task (58), they lack interpretability and demand greater computational power and time (71, 72). Computational resources are crucial during model deployment, given the higher prevalence of ACEs in disadvantaged communities that can benefit most from the models we developed (3, 37, 38, 73–76). Accordingly, we focus on classical ML models that can be scalable and adaptable, even in low-resource settings, while empowering end-users with explainable results to aid clinical decision-making.

## 2 Materials and methods

### 2.1 Data source

We utilized a subset of the latest publicly available data from the 2021 BRFSS (70). The BRFSS is a federally sponsored telephone-based survey conducted annually among U.S. adults. In 2021, the survey was conducted with 546,569 adults in all 50 states, the District of Columbia, and three territories in the U.S. The national survey collects data on SDoH, risky health behaviors, and the use of preventive services, among many other health-related factors, to facilitate health promotion efforts (70). Survey questions related to ACE exposure belong to an optional module of the BRFSS, which was implemented in 16 states in 2021 (Alabama, Arkansas, Iowa, Kansas, Maine, Mississippi, Nevada, New Hampshire, New Jersey, New York, Ohio, Oregon, South Carolina, Virginia, and Wisconsin). As ACE exposure was the study’s key predictor, our final dataset was limited to the data collected by these 16 states.

### 2.2 Inclusion criteria

Our inclusion criteria were individuals who (a) resided in any of the 16 U.S. states that administered the optional ACE module of the BRFSS, (b) answered all questions about ACEs, and (c) answered at least one of the questions regarding the pre-determined 13 chronic health conditions (*n* = 86,168). We excluded respondents with inconclusive responses (i.e., “Do not know/Not sure,” “Not Defined,” “Not asked,” “Yes, but female told only during pregnancy,” “Refused,” or missing answers) for any predictor and outcome variables (*n* = 32,900). As a result, our total sample size for analysis was 52,268 respondents.

### 2.3 Measures

The study’s outcome variables included 13 chronic health conditions (Supplementary Table S1). The predictor variables included self-reported ACE exposure, SDoH, health behaviors, and demographic and anthropometric characteristics (Supplementary Table S2). Please refer to Supplementary material for the answering options of each variable.

#### 2.3.1 Chronic health conditions

The outcome variables included self-reported diagnoses of 13 conditions with a well-established link to ACEs: (1) arthritis (including rheumatoid arthritis or other diseases with related symptoms, such as gout, lupus, or fibromyalgia), (2) asthma, (3) cancer (any type except skin cancer), (4) coronary heart disease (or angina), (5) depressive disorder (including depression, major depression, dysthymia, or minor depression), (6) pre-diabetes, (7) diabetes, (8) heart attack, (9) high blood pressure, (10) high cholesterol, (11) kidney disease, (12) pulmonary disease (chronic obstructive pulmonary disease, emphysema, or chronic bronchitis), and (13) stroke. These outcomes were categorized by the BRFSS as “Chronic Health Conditions” (77). Our final dataset included “Yes” and “No” responses.

#### 2.3.2 ACE exposure

ACE exposure was assessed with 11 questions on ACEs and two questions on positive childhood experiences (PCEs): (1) living with someone who was depressed, mentally ill, or suicidal (Yes/No); (2–3) two questions about living with someone who was a problem drinker or alcoholic or used illicit street drugs/abused prescription medications (Yes/No); (4) living someone who served time or was sentenced to serve time in prison or other correctional facility (Yes/No); (5) having parents who were separated or divorced (Yes/No/Parents Never Married); (6–8) three questions about living with parents who were physically and verbally abusive toward each other or the respondent (1 = “Never,” 2 = “Once,” 3 = “More than once”); (9–11) three questions on being sexually abused by an adult (1 = “Never,” 2 = “Once,” 3 = “More than once”); (12) the presence of an adult who made the respondent feel safe and protected; (13) the presence of an adult who ensured that the respondent’s basic needs were met. Both PCEs were evaluated on a 5-point Likert scale (1 = “Never,” 2 = “A little of the time,” 3 = “Some of the time,” 4 = “Most of the time,” 5 = “All of the time”), which were reverse-coded. Additionally, we computed two composite indices for ACE exposure: a binary variable measuring whether a respondent has experienced at least one ACE (Yes/No) and a numeric variable calculating the total number of ACEs experienced (range: 0–13).

#### 2.3.3 SDoH

The eight variables on SDoH included area of residence (urban vs. rural counties), education, employment status, income, renting/home ownership status, source of health insurance, availability of a personal healthcare provider, and inability to see a medical provider due to cost. These variables were categorical and had answering options unique to each question.

#### 2.3.4 Health behavior

The 13 variables included both health-promoting and deteriorating behaviors, such as exercise, smoking cigarettes, chewing tobacco, using e-cigarettes or vaping, heavy drinking, time since last cholesterol check, ever tested for HIV, monitoring blood sugar or blood pressure (two composite variables), cancer screening (two composite variables), and vaccination status (two composite variables). Like the SDoH, these variables were categorical and had differing rating scales.

We created six composite variables to handle missing data to preserve the information without dropping respondents: count of monitoring behaviors for blood sugar or blood pressure, ever monitored blood sugar or blood pressure, count of cancer screenings, ever screened for any cancer, count of vaccines received, and ever received any vaccines. The predictors for monitoring blood sugar or blood pressure were generated from two individual variables in the dataset (i.e., tested for blood sugar or diabetes in the past 3 years and regularly checked for blood pressure at home). These two variables were recoded, to where we assigned 1 (“Yes”) if the respondent checked their blood sugar or blood pressure and 0 (“No”) otherwise. The variable for the count of monitoring blood sugar or blood pressure was the sum of these binary items (range: 0–2).

Similarly, the cancer screening predictors were generated from six variables in the dataset (i.e., CT/CAT scan for lung cancer, mammogram for breast cancer, any cervical cancer screening, PSA test for prostate cancer, colonoscopy or sigmoidoscopy for colorectal cancer, and any other screening for colorectal cancer). These six variables were also re-engineered into binary variables (1 = “Yes,” 0 = “No”). The variable for the count of cancer screenings was the sum of their answers (range: 0–6). The variable measuring whether the respondent ever screened for any cancer was coded as 1 (“Yes”) if they underwent any of the six cancer screenings and 0 (“No”) if they underwent none.

Lastly, the vaccination status predictors were generated from five variables in the dataset (i.e., flu, pneumonia, tetanus, shingles, and zoster), which were re-engineered into binary variables (1 = “Yes,” 0 = “No”). The variable for the count of vaccines received was the sum of their answers (range: 0–5). The variable measuring whether the respondent ever received any vaccines was coded as 1 (“Yes”) if they received any of the five vaccines and 0 (“No”) if they received none.

#### 2.3.5 Demographic and anthropometric variables

Demographic variables included age (grouped in 13 five-year categories [1 = “18–24” to 13 = “80 ≤”]), race (White, Black, American Indian/Alaska Native, Asian, Native Hawaiian/Pacific Islander, Multiracial, Hispanic, Other), and sex (Male/Female). Body Mass Index (BMI) was the sole anthropometric variable available in the data and was assessed in four standard categories (1 = “Underweight,” 2 = “Normal Weight,” 3 = “Overweight,” and 4 = “Obese”).

### 2.4 Preprocessing

We recoded all variables (reverse coding as needed) on a 0-N scale, such that all “Never” and “No” variables were coded as zero. As noted previously, we excluded any respondents with “Do not know/Not sure,” “Refused,” “Not asked,” “Not defined,” and missing values for the outcome and predictor variables. In addition, we excluded variables for sexual orientation, transgender status, nutrition (i.e., consumption of fruits and vegetables and salt intake), and marijuana consumption in the last 30 days due to large volumes of missing data (*n* > 26,000 or roughly 50% of our data).

Moreover, given the data imbalance in our outcome variables (i.e., the proportion of respondents without chronic health conditions substantially exceeding their counterparts with such conditions), we performed random under-sampling of the majority class for each outcome by retaining the data for respondents with the chronic health conditions, and randomly dropping the data from the larger group without the conditions. This approach ensured equally sized classes for the outcome data, which could reduce the risk of model bias and computational burden (see Supplementary Figure S1). Relative to other sampling methods, random under-sampling is considered an effective approach to reducing data imbalance in sufficiently large datasets while minimizing the risk of generalization error on test data (78–80).

## 3 Data analysis

### 3.1 Univariate and bivariate

We conducted descriptive analyses (i.e., counts, percentages, mean, and standard deviation [SD]) for the predictor variables. Adopting Chi-square tests, we compared respondents with vs. without missing information to investigate any significant differences in their racial and income distributions and health outcomes and ultimately prevent potential biases that might be introduced into the final dataset by deleting the missing cases.

### 3.2 ML modeling

After random under-sampling, we split the data into training and test datasets. 80% of the data was allocated for training, while the remaining 20% was reserved for testing. We built a suite of supervised ML methods, such as LR, Gaussian NB, SVM, RF, and KNN, specific to each of our target chronic health conditions.

We evaluated model performance with accuracy (i.e., the rate of correct predictions) and Area under the Curve or AUC score (i.e., the probability of a model ranking a random positive observation higher than a random negative observation).

We performed hyperparameter tuning for each model on the training set to determine the most accurate predictors for each chronic health condition. Briefly, we tested a variety of optimization algorithms, penalty terms, and regularization strengths for LR; variance smoothing values for Gaussian NB; loss functions, penalty terms, and regularization strengths for SVM; the number of trees, number of features, maximum tree depth, and bootstrapping method for RF; the number of neighbors, weights, and distance metrics for KNN (see Supplementary Table S3 for more details). We utilized 3-fold cross-validation and evaluated performance using validation AUC score.

### 3.3 Model interpretation

We calculated the importance of each predictor variable in predicting the occurrence of each chronic health condition using different metrics for each ML model type (81). We then examined the variable importance of the best-performing model for each chronic health condition. We performed min-max normalization on each set of variable importances, converting them to 0–1 scales. This approach allowed us to compare relative variable importance across the models. We computed variable importance for each ML model type: for LR, we referred to the coefficients of the predictor variables in the regression formulation (80); for Gaussian NB, we employed permutation importance that measures the decline in model performance when individual random variables are shuffled (82); for SVM, we calculated the weight vector that represents the hyperplane separating the classes in linear space (83); for RF, we examined GINI importance or mean decrease in impurity, indicating how often a specific feature is selected for splitting within the RF and, thereby, its discriminative value toward the classification (84). We performed all procedures using Python 3.8.3 run on Jupyter Notebook. We used several open-source Python packages: *numpy, pandas, matplotlib, sci-kit learn, seaborn,* and *scipy.*

## 4 Results

### 4.1 Sample characteristics

As illustrated in Table 1, 39% of the respondents were aged 65 or older. About 83% of them self-identified as White and 8.4% as Black. Slightly over 50% were female and married, respectively. Over 40% of the respondents completed 4 years of college education or more and were employed, respectively, while 78% owned a home.

**Table 1:** Sample characteristics.

Demographic characteristics	
**Age, *n* (%)**	
18–24	1,826 (3.43)
25–29	1,947 (3.66)
30–34	2,648 (4.97)
35–39	3,258 (6.12)
40–44	3,579 (6.72)
45–49	3,673 (6.9)
50–54	4,416 (8.29)
55–59	5,019 (9.42)
60–64	6,086 (11.43)
65–69	6,429 (12.07)
70–74	6,104 (11.46)
75–79	3,944 (7.4)
>80	4,339 (8.15)
**Race/ethnicity, *n* (%)**	
White	44,155 (82.89)
Black	4,467 (8.39)
American Indian/Alaska Native	544 (1.02)
Asian	631 (1.18)
native Hawaiian/Pacific Islander	55 (0.10)
Other	346 (0.65)
Multiracial	823 (1.55)
Hispanic	2,247 (4.22)
**Sex, *n* (%)**	
Male	25,226 (47.36)
Female	28,042 (52.64)
**Marital status, *n* (%)**	
Married	30,000 (56.32)
Divorced	6,928 (13.01)
Widowed	6,107 (11.46)
Separated	963 (1.81)
Never married	7,533 (14.14)
Unmarried couple	1,737 (3.26)
**Anthropometric characteristics**	
**Body mass index, *n* (%)**	
Underweight	640 (1.2)
Normal	13,933 (26.16)
Overweight	19,089 (35.84)
Obese	19,606 (36.81)
**Social determinants of health (SDoH)**	
**Urban or rural county, *n* (%)**	
Urban counties	43,734 (82.10)
Rural counties	9,534 (17.90)
**Education level, *n* (%)**	
Never attended school	21 (0.04)
Grades 1–8	572 (1.07)
Grades 9–11	1,681 (3.16)
Grades 12—GED	13,046 (24.49)
College 1–3 years	15,173 (28.48)
College >4 years	22,775 (42.76)
**Employment status, *n* (%)**	
Employed for wages	23,396 (43.92)
Self-employed	4,475 (8.4)
Out of work for >1 year	1,042 (1.96)
Out of work for <1 year	868 (1.63)
Homemaker	1,715 (3.22)
Student	741 (1.39)
Retired	18,071 (33.92)
Unable to work	2,960 (5.56)
**Income, *n* (%)**	
< $10,000	1,127 (2.12)
$10,000–$15,000	1,585 (2.98)
$15,000–$20,000	2,077 (3.90)
$25,000–$30,000	3,107 (5.83)
$30,000–$35,000	6,626 (12.44)
$35,000–$50,000	7,742 (14.53)
$50,000–$75,000	9,594 (18.01)
$75,000–$100,000	7,820 (14.68)
$100,000–$150,000	7,759 (14.57)
$150,000–$200,000	3,120 (5.86)
>$200,000	2,711 (5.09)
**Rent or own home, *n* (%)**	
Own	41,687 (78.26)
Rent	10,009 (18.79)
Other arrangement	1,572 (2.95)
**Marital status, *n* (%)**	
Married	30,000 (56.32)
Divorced	6,928 (13.01)
Widowed	6,107 (11.46)
Separated	963 (1.81)
Never married	7,533 (14.14)
Unmarried couple	1,737 (3.26)
**Source of health insurance, *n* (%)**	
Employer or union plan	21,555 (40.47)
Private plan	4,342 (8.15)
Medicare	18,206 (34.18)
Medigap	59 (0.11)
Medicaid	2,500 (4.69)
Children’s Health Insurance Program (CHIP)	12 (0.02)
Military-related healthcare	1,845 (3.46)
Indian Health Service	60 (0.11)
State-sponsored health plan	1,136 (2.13)
Other government program	1,442 (2.71)
No coverage	2,111 (3.96)
**Has personal care provider, *n* (%)**	
Yes, only one	34,089 (64.0)
More than one	14,397 (27.03)
No	4,782 (8.98)
**Unable to see doctor due to medical cost, *n* (%)**	
Yes	3,180 (5.97)
No	50,088 (94.03)
**Health behavior**	
**Regular exercise, *n* (%)**	
Yes	40,412 (75.87)
No	12,856 (24.13)
**Smoked at least 100 cigarettes in life, *n* (%)**	
Yes	22,740 (42.69)
No	30,528 (57.31)
Currently use chewing tobacco, snuff or snuss, *n* (%)	
Every day	1,066 (2.0)
Some days	707 (1.33)
Not at all	51,495 (96.67)
**Use-cigarettes or electronic vaping products, *n* (%)**	
Every day	1,056 (1.98)
Some days	1,125 (2.11)
Not at all	43,338 (81.36)
Never used	7,749 (14.55)
**Heavy drinker, *n* (%)**	
No	49,893 (93.66)
Yes	3,375 (6.34)
**Time since last cholesterol check, *n* (%)**	
Never	3,739 (7.02)
<1 Year	39,096 (73.39)
1–2 Years	5,776 (10.84)
2–3 Years	1,803 (3.38)
3–4 Years	651 (1.22)
4–5 Years	580 (1.09)
>5 Years	1,623 (3.05)
**Ever tested for HIV, *n* (%)**	
Yes	17,195 (32.28)
No	36,073 (67.72)
**Count of monitoring behaviors for blood sugar and blood pressure, *n* (%)**	
0	33,238 (62.4)
1	18,837 (35.36)
2	1,193 (2.24)
**Count of cancer screenings, *n* (%)**	
0	49,771 (93.44)
1	2,354 (4.42)
2	1,095 (2.06)
3	48 (0.09)
**Count of vaccines received, *n* (%)**	
0	16,975 (31.87)
1	18,573 (34.87)
2	15,991 (30.02)
3	1,050 (1.97)
4	679 (1.27)
**ACE exposure**	
Four or more ACEs, *n* (%)	9,808 (18.41)
Total ACEs, mean (sd)	1.83 (2.27)

In terms of BMI, 35.8% of the respondents were overweight, and 36.8% were obese. For healthcare access, 64% reported having a personal provider, while 40.5% reported having an employer or union-sponsored insurance. Nevertheless, 94% reported that they could not see a doctor in the past 12 months due to cost. Regarding health behaviors, a majority of the respondents reported exercising in the past month (75.9%) and never using chewing tobacco (96.7%) and electronic cigarettes/vaping products (81.4%). Also, 57.3% had smoked less than 100 cigarettes in their lifetime, and around 6% were involved in heavy drinking.

73.4% of the respondents checked their cholesterol last time less than a year ago. On the other hand, a majority reported never having been tested for HIV (67.7%), not monitoring blood sugar or blood pressure (62.4%), and not screening for cancer (93.4%). Nearly one in three respondents received at least one vaccine. Lastly, the mean number of ACEs was 1.83 (SD = 2.27), and 18.4% of the respondents encountered four or more ACEs.

### 4.2 Analysis of missing data

There was no significant difference in the racial distribution of the missing and non-missing cases (data not shown). However, we found a significant difference in the income distribution between the two groups, wherein respondents with missing data were more likely to be in a higher-income group earning $75,000 or more. Regarding chronic health conditions, we found significant differences only in high blood pressure and arthritis, whereby those with missing data were more likely to experience these conditions. However, we do not expect the removal of missing data on high blood pressure and arthritis to impact model performance, as we performed under-sampling to ensure balanced distributions of classes for each outcome variable.

### 4.3 Model performance

With the inclusion of data on ACEs, our ML models achieved higher or similar accuracy and AUC scores compared to existing models in the literature that predicted health outcomes based on SDoH (65) (Table 2). Nine of the 13 models obtained test accuracies above 70% and test AUC scores above 0.7. The top-performing models were those predicting diabetes (78.4% accuracy, 0.784 AUC), pulmonary disease (75.3% accuracy, 0.753 AUC), and heart attack (73.2% accuracy, 0.732 AUC).

**Table 2:** Classification model performance for all target chronic health conditions.

Chronic disease	ML model	Validation accuracy [95% CI]	Test accuracy [95% CI]	Test AUC score [95% CI]
Arthritis *n* = 39,184	LR	0.696 [0.691, 0.7]	0.697 [0.693, 0.702]	0.697 [0.695, 0.7]
	NB	0.682 [0.678, 0.687]	0.688 [0.683, 0.693]	0.688 [0.685, 0.691]
	SVM	0.691 [0.686, 0.696]	0.697 [0.692, 0.701]	0.697 [0.694, 0.699]
	KNN	0.667 [0.662, 0.671]	0.677 [0.673, 0.682]	0.677 [0.675, 0.68]
	**RF (Best Predictor)**	**0.697 [0.693, 0.702]**	**0.701 [0.697, 0.706]**	**0.701 [0.699, 0.704]**
Asthma *n* = 14,268	LR	0.615 [0.607, 0.623]	0.615 [0.607, 0.623]	0.615 [0.61, 0.619]
	NB	0.572 [0.564, 0.58]	0.578 [0.57, 0.587]	0.578 [0.574, 0.583]
	SVM	0.615 [0.607, 0.623]	0.616 [0.608, 0.624]	0.616 [0.612, 0.621]
	KNN	0.569 [0.56, 0.577]	0.571 [0.563, 0.58]	0.571 [0.567, 0.576]
	**RF (Best Predictor)**	**0.61 [0.602, 0.618]**	**0.627 [0.619, 0.635]**	**0.627 [0.622, 0.631]**
Cancer *n* = 11,726	LR	0.675 [0.667, 0.684]	0.668 [0.66, 0.677]	0.668 [0.664, 0.673]
	NB	0.652 [0.644, 0.661]	0.661 [0.653, 0.67]	0.661 [0.656, 0.666]
	SVM	0.673 [0.665, 0.682]	0.671 [0.663, 0.68]	0.671 [0.667, 0.676]
	KNN	0.647 [0.639, 0.656]	0.659 [0.65, 0.668]	0.659 [0.654, 0.664]
	**RF (Best Predictor)**	**0.675 [0.666, 0.683]**	**0.687 [0.678, 0.695]**	**0.687 [0.682, 0.691]**
Coronary heart disease *n* = 6,554	LR	0.732 [0.721, 0.743]	0.715 [0.705, 0.726]	0.716 [0.709, 0.722]
	NB	0.704 [0.693, 0.715]	0.694 [0.683, 0.705]	0.694 [0.688, 0.701]
	**SVM (Best Predictor)**	**0.732 [0.721, 0.742]**	**0.725 [0.715, 0.736]**	**0.725 [0.719, 0.732]**
	KNN	0.7 [0.689, 0.711]	0.678 [0.667, 0.689]	0.678 [0.672, 0.685]
	RF	0.734 [0.724, 0.745]	0.719 [0.708, 0.729]	0.719 [0.712, 0.725]
Depressive disorder *n* = 21,288	**LR (Best Predictor)**	**0.708 [0.702, 0.714]**	**0.705 [0.699, 0.711]**	**0.705 [0.702, 0.709]**
	NB	0.648 [0.642, 0.655]	0.642 [0.636, 0.649]	0.642 [0.639, 0.646]
	SVM	0.705 [0.699, 0.711]	0.7 [0.694, 0.706]	0.7 [0.697, 0.704]
	KNN	0.665 [0.659, 0.671]	0.665 [0.659, 0.672]	0.665 [0.662, 0.669]
	RF	0.709 [0.703, 0.715]	0.702 [0.696, 0.709]	0.702 [0.699, 0.706]
Diabetes *n* = 15,504	LR	0.784 [0.778, 0.791]	0.772 [0.765, 0.778]	0.772 [0.768, 0.775]
	NB	0.764 [0.758, 0.771]	0.751 [0.744, 0.758]	0.751 [0.747, 0.755]
	SVM	0.783 [0.777, 0.79]	0.774 [0.768, 0.781]	0.774 [0.771, 0.778]
	KNN	0.737 [0.73, 0.744]	0.729 [0.722, 0.736]	0.729 [0.725, 0.733]
	**RF (Best Predictor)**	**0.79 [0.784, 0.797]**	**0.784 [0.778, 0.791]**	**0.784 [0.781, 0.788]**
Heart attack *n* = 6,236	LR	0.724 [0.713, 0.735]	0.725 [0.714, 0.736]	0.725 [0.719, 0.731]
	NB	0.695 [0.684, 0.707]	0.698 [0.687, 0.709]	0.698 [0.691, 0.704]
	SVM	0.722 [0.711, 0.733]	0.725 [0.714, 0.736]	0.725 [0.719, 0.731]
	KNN	0.689 [0.678, 0.701]	0.691 [0.679, 0.702]	0.691 [0.684, 0.697]
	**RF (Best Predictor)**	**0.728 [0.717, 0.739]**	**0.732 [0.721, 0.743]**	**0.732 [0.726, 0.739]**
High blood pressure *n* = 45,976	LR	0.707 [0.703, 0.711]	0.712 [0.708, 0.717]	0.712 [0.71, 0.715]
	NB	0.669 [0.665, 0.673]	0.669 [0.665, 0.673]	0.669 [0.667, 0.672]
	SVM	0.707 [0.703, 0.711]	0.711 [0.707, 0.715]	0.711 [0.709, 0.714]
	KNN	0.676 [0.672, 0.68]	0.679 [0.674, 0.683]	0.679 [0.676, 0.681]
	**RF (Best Predictor)**	**0.713 [0.709, 0.717]**	**0.716 [0.711, 0.72]**	**0.716 [0.713, 0.718]**
High cholesterol *n* = 49,526	LR	0.656 [0.652, 0.66]	0.657 [0.653, 0.662]	0.657 [0.655, 0.66]
	NB	0.612 [0.607, 0.616]	0.613 [0.608, 0.617]	0.613 [0.61, 0.615]
	SVM	0.66 [0.656, 0.664]	0.661 [0.657, 0.665]	0.661 [0.658, 0.663]
	KNN	0.609 [0.605, 0.613]	0.612 [0.608, 0.617]	0.612 [0.61, 0.615]
	**RF (Best Predictor)**	**0.67 [0.666, 0.675]**	**0.671 [0.667, 0.675]**	**0.671 [0.668, 0.673]**
Kidney disease *n* = 4,432	LR	0.692 [0.678, 0.706]	0.677 [0.663, 0.69]	0.677 [0.669, 0.685]
	NB	0.679 [0.665, 0.692]	0.653 [0.639, 0.667]	0.653 [0.645, 0.661]
	SVM	0.691 [0.678, 0.705]	0.669 [0.655, 0.683]	0.669 [0.661, 0.677]
	KNN	0.674 [0.66, 0.688]	0.633 [0.619, 0.647]	0.633 [0.625, 0.641]
	**RF (Best Predictor)**	**0.698 [0.684, 0.711]**	**0.681 [0.667, 0.695]**	**0.681 [0.673, 0.689]**
Pre-diabetes *n* = 51,060	LR	0.711 [0.707, 0.715]	0.717 [0.714, 0.721]	0.714 [0.712, 0.717]
	NB	0.694 [0.69, 0.698]	0.701 [0.697, 0.705]	0.696 [0.694, 0.699]
	SVM	0.708 [0.704, 0.712]	0.715 [0.711, 0.719]	0.711 [0.709, 0.713]
	KNN	0.671 [0.667, 0.675]	0.679 [0.675, 0.684]	0.674 [0.671, 0.676]
	**RF (Best Predictor)**	**0.724 [0.72, 0.728]**	**0.728 [0.724, 0.732]**	**0.726 [0.724, 0.728]**
Pulmonary disease *n* = 8,890	**LR (Best Predictor)**	**0.745 [0.736, 0.754]**	**0.753 [0.744, 0.762]**	**0.753 [0.748, 0.758]**
	NB	0.711 [0.701, 0.72]	0.715 [0.706, 0.725]	0.715 [0.71, 0.721]
	SVM	0.745 [0.736, 0.754]	0.749 [0.74, 0.758]	0.749 [0.744, 0.754]
	KNN	0.706 [0.697, 0.716]	0.711 [0.701, 0.72]	0.711 [0.706, 0.716]
	RF	0.756 [0.747, 0.765]	0.744 [0.734, 0.753]	0.744 [0.738, 0.749]
Stroke *n* = 4,488	LR	0.714 [0.701, 0.728]	0.714 [0.701, 0.727]	0.714 [0.706, 0.721]
	NB	0.706 [0.693, 0.72]	0.688 [0.675, 0.702]	0.688 [0.68, 0.696]
	SVM	0.712 [0.698, 0.725]	0.71 [0.697, 0.724]	0.71 [0.703, 0.718]
	KNN	0.698 [0.685, 0.712]	0.682 [0.668, 0.695]	0.682 [0.674, 0.689]
	**RF (Best Predictor)**	**0.718 [0.705, 0.731]**	**0.715 [0.702, 0.728]**	**0.715 [0.707, 0.722]**

Bold values indicate best-performing models for each chronic disease.

Training a single iteration of each model took an average of 38 s. Validation and model selection involved training a single iteration of each algorithm for every combination of the hyperparameters that were tested. This process determined the optimal performance for each model.

Three of the top five models employed RF (diabetes, heart attack, and prediabetes), whereas LR (pulmonary disease) and SVM (coronary heart disease) were used in the other two. Overall, RF performed best for 10 of the 13 chronic health conditions: diabetes, heart attack, prediabetes, high blood pressure, stroke, arthritis, cancer, kidney disease, high cholesterol, and asthma. The linear model (i.e., LR) performed best only for two chronic health conditions.

### 4.4 Model interpretation

Age and SDoH, such as income, employment, and health insurance, were among the top five strongest variables to predict each chronic health condition (Table 3). ACEs, either cumulatively or individually, were also identified as an important variable for asthma, coronary heart disease, depressive disorder, and pulmonary disease. When individually examined, living with a mentally ill/suicidal person during childhood was the only ACE predictive of these health conditions (except asthma). Specifically, living with a mentally ill/suicidal person seemed to play the most critical role in the depressive disorder and coronary heart disease models and was listed as their first and second most important predictor, respectively. Supplementary Figure S2 outlines the variable importance of all models.

**Table 3:** Most predictive variables for each best-performing classification model.

Disease	Best performing model	Top 5 predictor variables
Arthritis	Random forest	Age
		Employment status
		Income
		Source of health insurance
		Count of vaccines received
Asthma	Random forest	Age
		Income
		Total ACEs
		BMI category
		Employment status
Cancer	Random forest	Age
		Employment status
		Source of health insurance
		Income
		Count of vaccines received
Coronary heart disease	Support vector machine	Sex
		ACE—lived with mentally ill/suicidal person
		Has personal health care provider available
		Smoking cigarettes
		Age
Depressive disorder	Logistic regression	ACE—lived with mentally ill/suicidal person
		Inability to see medical provider due to cost
		Sex
		Exercise
		Ever received any vaccines
Diabetes	Random forest	Age
		Ever monitored blood sugar or blood pressure
		Count of blood monitoring behaviors for blood sugar or blood pressure
		BMI
		Time since last cholesterol check
Heart attack	Random forest	Age
		Employment status
		Income
		Source of health insurance
		Sex
High blood pressure	Random forest	Age
		BMI
		Employment status
		Time since last cholesterol check
		Income
High cholesterol	Random forest	Time since last cholesterol check
		Age
		Income
		Employment status
		Source of health insurance
Kidney disease	Random forest	Employment status
		Age
		Source of health insurance
		Income
		Count of vaccines received
Pre-diabetes	Random forest	Ever monitored blood sugar or blood pressure
		Count of blood monitoring behaviors for blood sugar or blood pressure
		Age
		Income
		Time since last cholesterol check
Pulmonary disease	Logistic regression	Smoking cigarettes
		Exercise
		Inability to see medical provider due to cost
		Have you tested for HIV?
		ACE—lived with mentally ill/suicidal person
Stroke	Random forest	Age
		Employment status
		Income
		Source of health insurance
		Time since last cholesterol check

**Figure 1:**
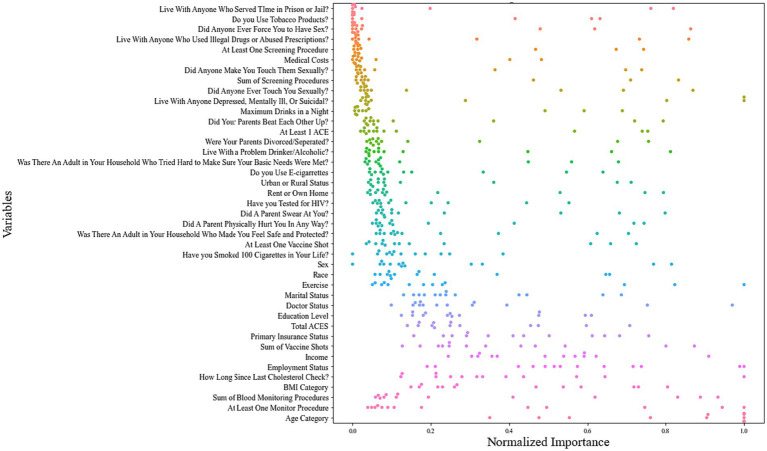
Swarm plot of normalized variable importance across all 13 chronic health condition models.

Normalized variable importance revealed the 10 most predictive variables across a total of 65 models (5 ML models × 13 chronic health conditions): age, ever monitored blood sugar or blood pressure, count of monitoring behaviors for blood sugar or blood pressure, BMI, time since last cholesterol check, employment status, income, count of vaccines received, primary insurance status, and the total number of ACEs (Figure 1).

## 5 Discussion

Our study developed explainable ML models using large national survey data to predict 13 chronic health conditions prevalent among U.S. adults. We found that non-linear models, particularly RF, outperformed the linear model in predicting chronic health conditions. In addition, our ML models cast light on the most predictive features of each condition. Among these, ACEs and SDoH such as income, employment, and health insurance, were robust predictors of multiple chronic health conditions. Additionally, cumulative ACEs were a stronger predictor than individual ACEs across chronic health conditions. Our models achieved comparable or superior performance to classical ML-based health outcome prediction models that previously used SDoH as predictors (65). Our findings not only align with previous studies linking ACEs to chronic health conditions (6–28, 30, 31), but also expand upon them by employing ML to factor in complex interactions between ACEs and other socioeconomic and behavioral factors to predict chronic health conditions. Our primary focus on ACEs and relevant socioeconomic and behavioral factors can distinguish the current study from others. While previous studies have documented excellent performance of classical ML models (e.g., RF, gradient boost, SVM, LR, KNN, decision trees, and NB) to predict chronic health conditions, they commonly focused on biomedical predictors such as clinical, biomarker, and genetics data (58, 60, 63, 64).

Our study, which emphasizes the role of ACEs and their cumulative impact, highlights the significance of predictive values of total ACEs in shaping chronic diseases. ACEs were among the top five predictors for four chronic health conditions: asthma, coronary heart disease, depressive disorder, and pulmonary disease. Living with a mentally ill/suicidal person during childhood was particularly predictive of coronary heart disease, depressive disorder, and pulmonary disease. These results are supported by Gallagher and colleagues, who found that living with a severely mentally ill person is associated with poorer subjective health, activity limitations, and higher utilization of physician visits than living with non-mentally ill household members (85). Beyond this single ACE, the total number of ACEs was a stronger predictor than individual ACEs across all the best-performing models of the 13 chronic health conditions. Notably, the total number of ACEs was among the top five predictors for the asthma model, which aligns with findings from the existing literature on the dose–response relationship between ACEs and asthma (12). The composite measure may more accurately represent how ACEs operate: not arbitrarily, but rather in clusters, especially among historically marginalized populations (2, 86–88). This finding underscores the significance of cumulative ACEs on an individual’s likelihood of developing chronic health conditions. Although we demonstrated a strong association between ACEs and chronic health conditions by comparing various base learners, including LR, Gaussian NB, SVM, RF, and KNN, future work is guaranteed to improve prediction accuracy. For example, we may employ stacked ensemble algorithms (e.g., XGBoost), which has been reported to improve classification with imbalanced data (89–91); this may enhance performance while requiring smaller degrees of undersampling, thereby allowing the use of a larger volume of data. Additionally, we may perform more extensive iterations of training and validation using a wider range of hyperparameters.

On a relative scale, the models for diabetes, pulmonary disease, and heart attacks performed particularly well, whereas models predicting kidney disease, high cholesterol, and asthma exhibited lower performance. Such discrepancies may be attributable to the varying importance of different variables in predicting distinct chronic health conditions. Similarly, Battineni and colleagues report that their ML models used different sets of variables to predict various chronic diseases in different populations, demonstrating no “gold standard” for ML methods to predict chronic diseases, including how to select and prioritize predictors (56). Despite improved interpretability, this unclarity could still compromise ML models’ transparency and trustworthiness. To partially address the issue, future research could compare different sets of predictors across domains, ML models, and strategies for interpretability to analyze the commonalities and variations in model output.

In addition to the ACEs discussed above, the following were the most predictive variables across all models of chronic health conditions: age, ever monitored blood sugar or blood pressure, count of monitoring behaviors for blood sugar or blood pressure, BMI, time since last cholesterol check, employment status, income, vaccine count, and primary health insurance status. Previous literature has revealed that chronic health conditions are indeed associated with age (92); self-management (93, 94); BMI (95); employment, income, and wealth (96–98); immunization (99–103); health insurance (104, 105).

Our study findings undergird the pivotal role of preventing ACEs and socioeconomic inequalities in chronic disease prevention at the population level. Our ML models could enable data-driven screening for various chronic health conditions to identify high-risk individuals, explain the most influential underlying factors, and develop personalized prevention strategies.

Despite the strengths and contributions of our study, some limitations must be acknowledged. First, we analyzed self-reported data, which could have introduced biases (e.g., recall bias, social desirability bias, or misinterpretation of the questions), potentially affecting the accuracy and reliability of the developed models. However, such reporting biases are inherent in survey data and not unique to the BRFSS. In addition, the prevalence estimates in the BRFSS data are known to be consistent with comparable national surveys (i.e., National Health Interview Survey, National Health and Nutrition Examination Survey) (106, 107). More objective measures, such as biomarkers, should be analyzed to predict chronic health conditions more accurately in the future.

Second, our final dataset comprised mostly White and middle-income respondents. Consequently, the developed models may not predict chronic health conditions among disadvantaged populations at higher risk of ACEs (e.g., Black, Hispanic, or low-income individuals) as accurately as among more privileged populations (e.g., White or affluent individuals). Future studies are needed to develop ML models optimized for subpopulations, compare their performance to models with a pooled population, and consider potential differences in important variables or magnitudes in prediction. Stratification by subpopulation could partially mitigate the system-wide bias in collecting and processing data among different populations.

Third, our random sampling method to create an artificially balanced dataset for model training may misrepresent model performance. Random under-sampling increases the possibility that the model underperforms with “real-world” data, as the inflated proportion of positive cases in the training data may introduce greater false positives in real-world data. However, relative to other sampling methods, random under-sampling minimizes the risk of generalization error on test data (78–80).

Fourth, we encountered some hurdles with data availability. For instance, there were no core questions in the BRFSS regarding transportation, food security, and green space, which are crucial SDoH. Relatedly, other variables that represent determinants of health were not factored into our models due to insufficient data: sexual orientation, transgender status, nutrition, and marijuana consumption. Furthermore, we were unable to predict specific types of cancer, joint conditions, and pulmonary disease due to unavailable data.

Lastly, our ML models were trained and tested on unweighted data due to a lack of computing resources to model the weighted data. Hence, our unweighted ML models are limited in generalizability, and their performance is likely inflated to some degree compared to weighted models (108). With these limitations in data collection and modeling, our findings should be interpreted with caution. Our models should be viewed as supplementary tools for screening and decision-making, rather than a standalone, definitive prediction system for chronic health conditions.

## 6 Conclusion

To our knowledge, this is the first study to employ interpretable ML methods to model the syndemic interactions of ACEs, SDoH, health behaviors, and chronic health conditions using extensive data from a large national health survey in the U.S. Our findings highlighted the significance of preventing ACEs and mitigating their cumulative impact on chronic health conditions later in life. This study serves as an initial step toward developing a data-driven screening tool to identify U.S. adults at high risk of chronic health conditions, aiding in prevention and early intervention efforts. Our models also offer an interpretable and trauma-informed framework, aimed at reducing the persistent inequalities associated with early trauma and chronic health conditions among U.S. adults. Acknowledging the insights from Battineni et al., we underscore the importance of continuous validation and testing of our models to ensure their reliability and practical utility in multiple settings with different patient characteristics. ML models are bound to the data they train; therefore, the model parameters we have developed can be used as a baseline, upon which future research can develop contextualized models that will be re-fitted to other datasets of new patient populations to predict their chronic health conditions more accurately.

## Data availability statement

Publicly available datasets were analyzed in this study. This data can be found at: https://www.cdc.gov/brfss/annual_data/annual_2021.html.

## Ethics statement

Ethical approval was not required for the study involving humans in accordance with the local legislation and institutional requirements. Written informed consent to participate in this study was not required from the participants or the participants’ legal guardians/next of kin in accordance with the national legislation and the institutional requirements.

## Author contributions

HA: Data curation, Formal analysis, Investigation, Methodology, Resources, Software, Writing – original draft. TJ: Conceptualization, Data curation, Methodology, Project administration, Software, Writing – original draft. YM: Visualization, Writing – original draft. AM: Visualization, Writing – original draft. AS: Validation, Writing – review & editing. SK: Supervision, Validation, Writing – review & editing.
