# Supplementary material for: Can adverse childhood experiences predict chronic health conditions? Development of trauma-informed, explainable machine learning models
Source: Front Public Health. 2024 Jan 15;11:1309490. doi: 10.3389/fpubh.2023.1309490 (PMC10851779; doi:10.3389/fpubh.2023.1309490)
Supplement: Supplementary file 1 [file Data_Sheet_1.docx]

Supplementary Material

# Supplementary Figures and Tables

**Supplementary Table S1**. Information on outcome variables used from the BRFSS.

| **Variable Name** | **Question/Description** | **Value Labels**** |
| --- | --- | --- |
| BPHIGH6 | Have you ever been told by a doctor, nurse or other health professional that you have high blood pressure? | 1: Yes 3: No |
| TOLDHI3 | Have you ever been told by a doctor, nurse or other health professional that your cholesterol is high? | 1: Yes 2: No |
| CVDINFR4 | Ever told you had a heart attack, also called a myocardial infarction? | 1: Yes 2: No |
| CHCOCNCR | Ever told you had any other types of cancer? [Outside of skin cancer] | 1: Yes 2: No |
| CVDCRHD4 | Ever told you had angina or coronary heart disease? | 1: Yes 2: No |
| CVDSTRK3 | Ever told you had a stroke? | 1: Yes 2: No |
| ASTHMA3 | Ever told you had asthma? | 1: Yes 2: No |
| CHCCOPD3 | Ever told you had C.O.P.D. (chronic obstructive pulmonary disease), emphysema or chronic bronchitis? | 1: Yes  2: No |
| ADDEPEV3 | Ever told you had a depressive disorder (including depression, major depression, dysthymia, or minor depression)? | 1: Yes  2: No |
| CHCKDNY2 | Not including kidney stones, bladder infection or incontinence, were you ever told you had kidney disease? | 1: Yes  2: No |
| DIABETE4 | Ever told you had diabetes? | 1: Yes 2: No |
| HAVARTH5 | Has a doctor, nurse or other health professional ever told you that you had some form of arthritis, rheumatoid arthritis, gout, lupus, or fibromyalgia? (arthritis diagnoses include: rheumatism, polymyalgia rheumatic, osteoarthritis (not osteoporosis), tendonitis, bursitis, bunion, tennis elbow, carpal tunnel syndrome, tarsal tunnel syndrome, joint infection, Reiter’s syndrome, ankylosing spondylitis) | 1: Yes  2: No |
| PREDIAB1 | Have you ever been told by a doctor or other health professional that you have pre-diabetes or borderline diabetes? | 1: Yes 2: No |

****** For easier referencing, variables obtained directly from the BRFSS are displayed in the table with their original value labels. However, during data preprocessing, all variables were recoded as 0 (no) or 1 (yes). “Yes, but female told only during pregnancy,” “Don’t know/Not sure,” “Refused,” “Not asked,” “Not defined,” and “Missing” values were dropped.

**Supplementary Table S2.** Information on predictor variables used from the BRFSS.

| **Category** | **Variable Name*** | **Question/Description** | **Value Labels**** |
| --- | --- | --- | --- |
| Demographic | _AGEG5YR | Age in five-year categories | 1: 18-24 2: 25-29 3: 30-34 4: 35-39 5: 40-44 6: 45-49 7: 50-54 8: 55-59 9: 60-64 10: 65-69 11: 70-74 12: 75-79 13: 80 ≤ |
| Demographic | _RACE | Race/ethnicity categories | 1: White only, non-Hispanic 2: Black only, non-Hispanic 3: American Indian or Alaskan Native only, non-Hispanic 4: Asian only, non-Hispanic 5: Native Hawaiian or other Pacific Islander only, Non-Hispanic 6: Other race only, non-Hispanic 7: Multiracial, non-Hispanic 8: Hispanic |
| Demographic | _SEX | Sex | 1: Male 2: Female |
| Demographic | MARITAL | Marital Status | 1: Married 2: Divorced 3: Widowed 4: Separated 5: Never married 6: A member of an unmarried couple |
| Anthropometric | _BMI5CAT | Four-categories of Body Mass Index (BMI) | 1: underweight 2: normal weight 3: overweight 4: obese |
| Social determinant of health | _URBSTAT | Urban/Rural Status | 1: Urban counties 2: Rural Counties |
| Social determinant of health | EDUCA | What is the highest grade or year of school you completed? | 1: Never attended school or only kindergarten 2: Grades 1 through 8 (Elementary) 3: Grades 9 through 11 (Some high school) 4: Grade 12 or GED (High school graduate)  5: College 1 year to 3 years (Some college or technical school) 6: College 4 years or more (College graduate) |
| Social determinant of health | EMPLOY1 | Employment Status | 1: Employed for wages 2: Self-employed 3: Out of work for 1 year or more 4: Out of work for < 1 year 5: A homemaker 6: A student 7: Retired 8: Unable to work |
| Social determinant of health | INCOME3 | Is your annual household income from all sources: | 1: ≤$10,000 2: $10,000 to < $15,000 3: $15,000 to < $20,000 4: $20,000 to < $25,000 5: $25,000 to < $35,000 6: $35,000 to < $50,000 7: $50,000 to < $75,000 8: $75,000 to < $100,000 9: $100,000 to < $150,000 10: $150,000 to < $200,000 11: $200,000 ≤ |
| Social determinant of health | RENTHOM1 | Do you own or rent your home? | 1: Own 2: Rent 3: Other arrangement |
| Social determinant of health | PRIMINSR | What is the current primary source of your health insurance? | 1: A plan purchased through an employer or union (including plans purchased through another person´s employer) 2: A private nongovernmental plan that you or another family member buys on your own 3: Medicare 4: Medigap 5: Medicaid 6: Children´s Health Insurance Program (CHIP) 7: Military related healthcare: TRICARE/VA health care/CHAMP-VA 8: Indian Health Service 9: State sponsored health insurance 10: Other government program 88: No coverage of any type |
| Social determinant of health | PERSDOC3 | Do you have one person (or a group of doctors) that you think of as your personal health care provider? | 1: Yes, only one 2: More than one 3: No |
| Social determinant of health | MEDCOST1 | Was there a time in the past 12 months when you needed to see a doctor but could not because you could not afford it? | 1: Yes 2: No |
| Health behavior | EXERANY2 | During the past month, other than your regular job, did you participate in any physical activities or exercises such as running, calisthenics, golf, gardening, or walking for exercise? | 1: Yes 2: No |
| Health behavior | SMOKE100 | Have you smoked at least 100 cigarettes in your entire life? [Note: 5 packs = 100 cigarettes] | 1: Yes 2: No |
| Health behavior | USENOW3 | Do you currently use chewing tobacco, snuff, or snus every day, some days, or not at all? | 1: Yes 2: No 3: Not at all |
| Health behavior | ECIGNOW1 | Do you now use e-cigarettes or other electronic vaping products every day, some days, or not at all? | 1: Every day 2: Some days 3: Not at all 4: Never used e-cigs |
| Health behavior | _RFDRHV7 | Alcohol consumption calculated variable. Heavy drinkers (adult men having more than 14 drinks per week and adult women having more than 7 drinks per week | 1: No  2: Yes |
| Health behavior | CHOLCHK3 | About how long has it been since you last had your cholesterol checked? | 1: Never 2: Within the past year (anytime < one year ago) 3: Within the past 2 years (1 year but < 2 years ago) 4: Within the past 3 years (2 year but < 3 years ago) 5: Within the past 4 years (3 year but < 4 years ago) 6: Within the past 5 years (4 year but < 5 years ago) 8: 5 or more years ago |
| Health behavior | HIVTST7 | Including fluid testing from your mouth, but not including tests you may have had for blood donation, have you ever been tested for HIV? | 1: Yes 2: No |
| Health behavior | summonitor^+^ (PDIABTST, HOMRGCHK) | Combined count of the monitoring behaviors for blood sugar or blood pressure.   Includes: - testing for high blood sugar or diabetes in the past 3 years  - regularly checking for blood pressure at home  Both variables were binarized (yes/no), and the binary values were summed. | 0-2. |
| Health behavior | monitorpresent^+^ (PDIABTST, HOMRGCHK) | Ever monitored blood sugar or blood pressure. | 0: No 1: Yes |
| Health behavior | sumscreen^+^  (LCSCTSCN, HADMAM, CERVSCRN, PSATEST1, HADSIGM4, COLONCNCR) | Combined count of cancer screenings. Includes: - CT/CAT scan for lung cancer - mammogram for breast cancer - any cervical cancer screening - PSA test for prostate cancer  - colonoscopy or sigmodioscopy for colorectal cancer - any other screening for colorectal cancer  Due to inconsistent rating scales, each variable was binarized (yes/no), and the binary values were summed. | 0-6 |
| Health behavior | screenpresent^+^  (LCSCTSCN, HADMAM, CERVSCRN, PSATEST1, HADSIGM4, COLONCNCR) | Ever screened for any cancer. | 0: No 1: Yes |
| Health behavior | sumshots^+^  (FLUSHOT7,  PNEUVAC4,  HPVADC4,  TETANUS1,  SHINGLE2) | Combined count of immunizations received. Includes variables on: - receiving an adult flu shot/flu spray in the past 12 months - ever receiving a pneumonia vaccine  - ever receiving an HPV vaccine - receiving a tetanus shot in the past 10 years - ever receiving a shingles or zoster vaccine  Due to inconsistent rating scales, each value was binarized (yes/no), and the binary values were summed. | 0-5 |
| Health behavior | shotpresent^+^ | Ever received any vaccine | 0: No 1: Yes |
| ACE exposure | ACEDEPRS | Did you live with anyone who was depressed, mentally ill, or suicidal? | 1: Yes 2: No |
| ACE exposure | ACEDRINK | Did you live with anyone who was a problem drinker or alcoholic? | 1: Yes 2: No |
| ACE exposure | ACEDRUGS | Did you live with anyone who used illegal street drugs or who abused prescription medications? | 1: Yes 2: No |
| ACE exposure | ACEPRISN | Did you live with anyone who served time or was sentenced to serve time in a prison, jail, or other correctional facility? | 1: Yes 2: No |
| ACE exposure | ACEDIVRC | Were your parents separated or divorced? | 1: Yes 2: No  8: Parents never married |
| ACE exposure | ACEPUNCH | How often did your parents or adults in your home ever slap, hit, kick, punch or beat each other up? | 1: Never 2: Once 3: More than once |
| ACE exposure | ACEHURT1 | Not including spanking, (before age 18), how often did a parent or adult in your home ever hit, beat, kick, or physically hurt you in any way? Was it— | 1: Never 2: Once 3: More than once |
| ACE exposure | ACESWEAR | How often did a parent or adult in your home ever swear at you, insult you, or put you down? | 1: Never 2: Once 3: More than once |
| ACE exposure | ACETOUCH | How often did anyone at least 5 years older than you or an adult, ever touch you sexually? | 1: Never 2: Once 3: More than once |
| ACE exposure | ACETTHEM | How often did anyone at least 5 years older than you or an adult, try to make you touch them sexually? | 1: Never 2: Once 3: More than once |
| ACE exposure | ACEHVSEX | How often did anyone at least 5 years older than you or an adult, force you to have sex? | 1: Never 2: Once 3: More than once |
| ACE exposure | ACEADSAF | For how much of your childhood was there an adult in your household who made you feel safe and protected? Would you say never, a little of the time, some of the time, most of the time, or all of the time? | 1: Never 2: A little of the time 3: Some of the time 4: Most of the time 5: All of the time |
| ACE exposure | ACEADNED | For how much of your childhood was there an adult in your household who tried hard to make sure your basic needs were met? Would you say never, a little of the time, some of the time, most of the time, or all of the time? | 1: Never 2: A little of the time 3: Some of the time 4: Most of the time 5: All of the time |
| ACE exposure | sumace | Combined number of ACEs. Computed from ACE variables. Due to inconsistent rating scales, recoded to yes/no binary variables and summed. | 0-13 |
| ACE exposure | acepresent | Presence of any ACE. Computed from ACE variables | 0: No 1: Yes |

*Capitalized variable names were obtained directly from the BRFSS. Variable names in lowercase were computed by the authors of the study.
** For easier referencing, variables obtained directly from the BRFSS are displayed in the table with their original value labels. However, during data preprocessing, all variables were recoded (and reverse coded as needed) on a 0-N scale, such that all “never” and “no” variables were coded as zero. “Don’t know/Not sure,” “Refused,” “Not asked,” “Not defined,” and “Missing” values were dropped.

^+^ Computed to account for a large number of missing values in individual variables.

**Supplementary Table S3.** Hyperparameters Tested

| **Model** | **Hyperparameter** | **Values Tested** |
| --- | --- | --- |
| Logistic Regression | Solver | Newton, LBFGS, Lib-Linear |
|  | Penalty | L2 |
|  | C-Value | 100, 10, 1, 0.1, 0.01 |
| Gaussian Naïve Bayes | Variable Smoothing | Evenly spaced logarithmic scale from -9 to 0 |
| Support Vector Machine | Loss Function | Hinge, Squared Hinge |
|  | Penalty | L1, L2 |
|  | C-Value | 100, 10, 1, 0.1 |
| K-Nearest Neighbor | Number of Neighbors | 5, 7, 9, 11 |
|  | Weights | Uniform, Distance |
|  | Distance Metric | Minkowski, Euclidean, Manhattan |
| Random Forest | Number of Estimators | 200, 400, 600, 800, 1000 |
|  | Maximum Feature Split | Auto, Square Root |
|  | Maximum Depth | 5, 28, 52, 76, 100 |
|  | Bootstrap | True, False |

## Supplementary Figures


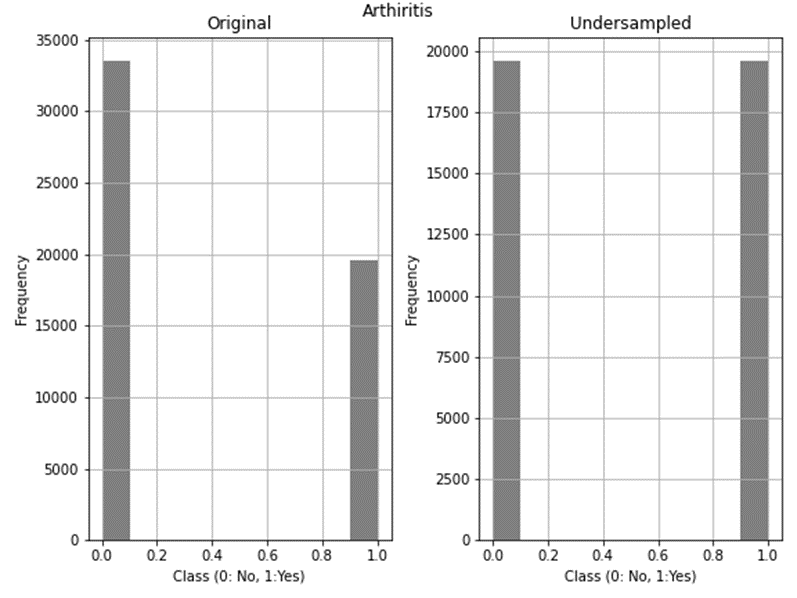


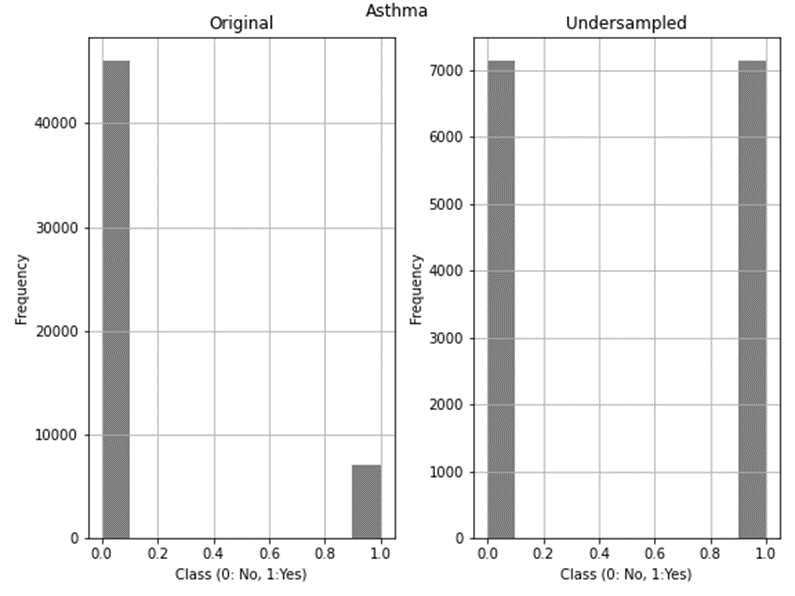


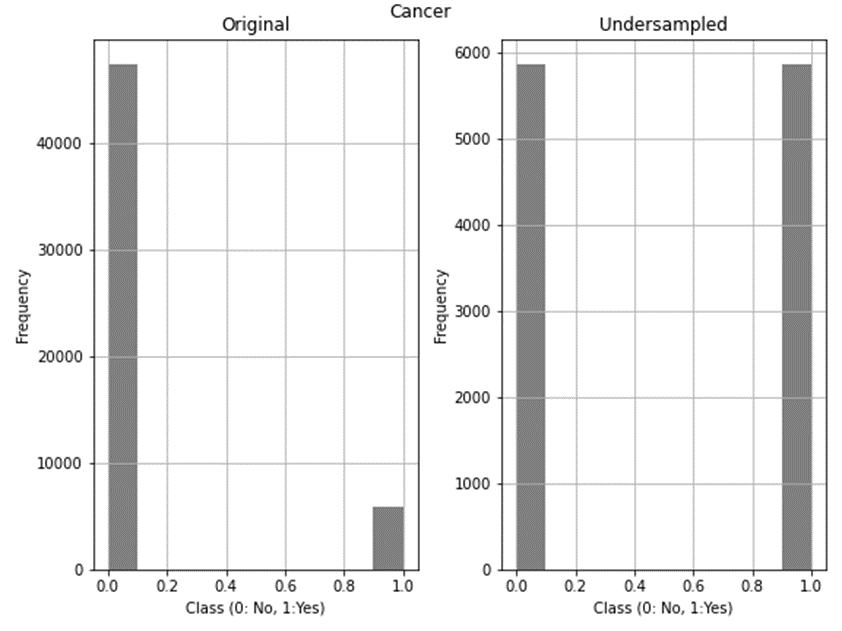


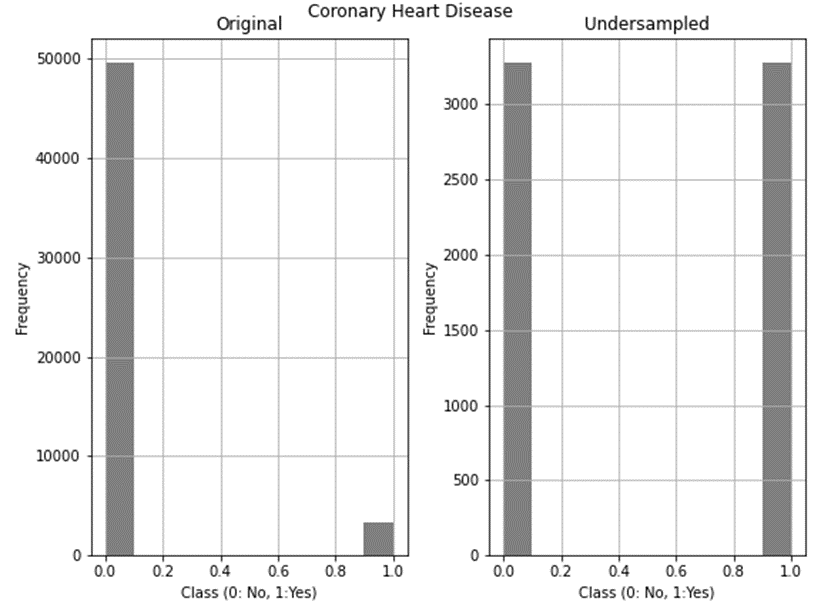


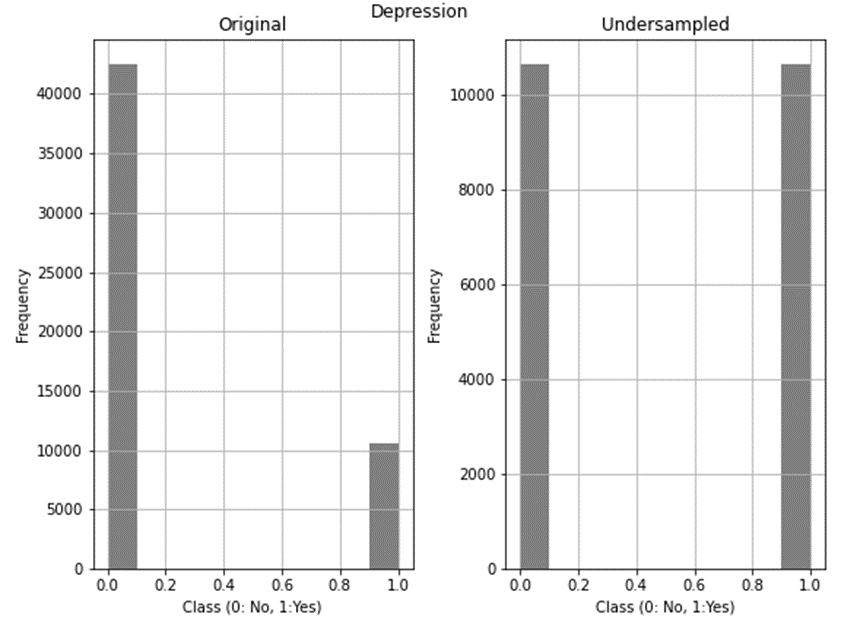


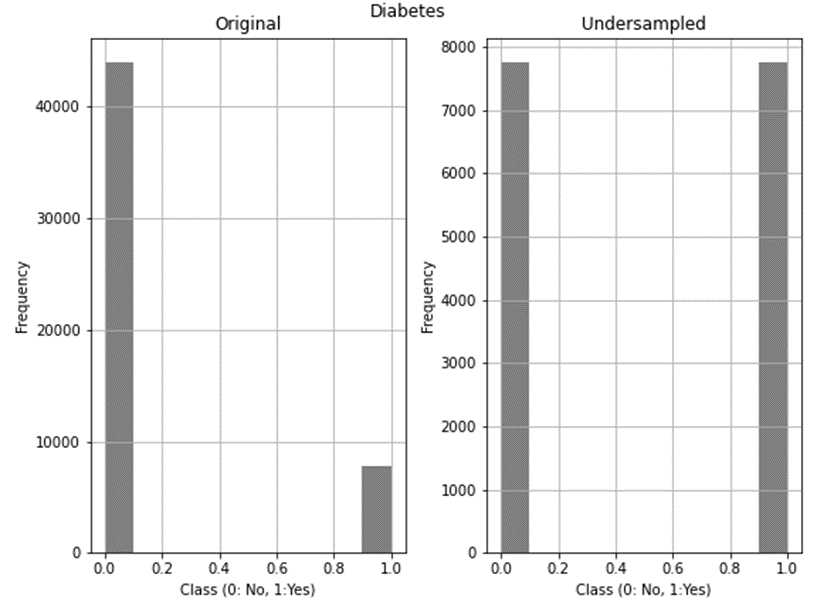


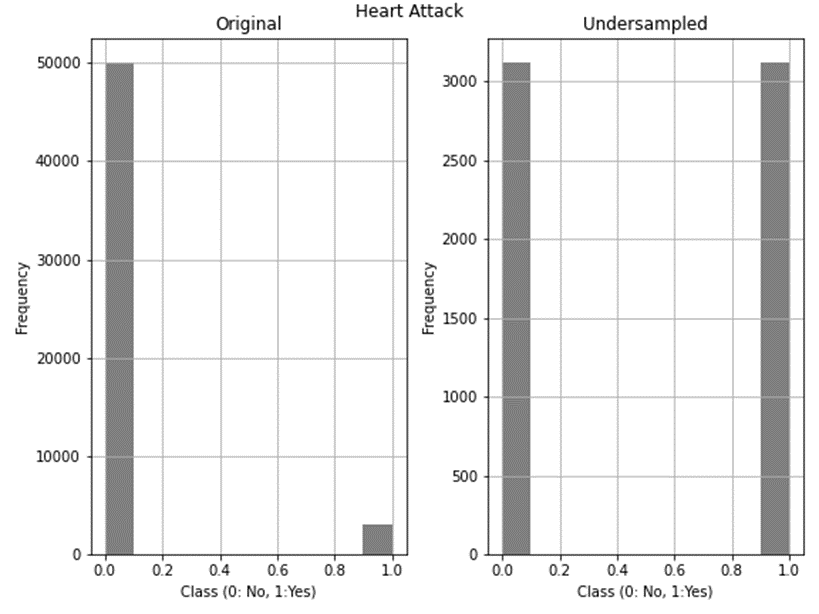


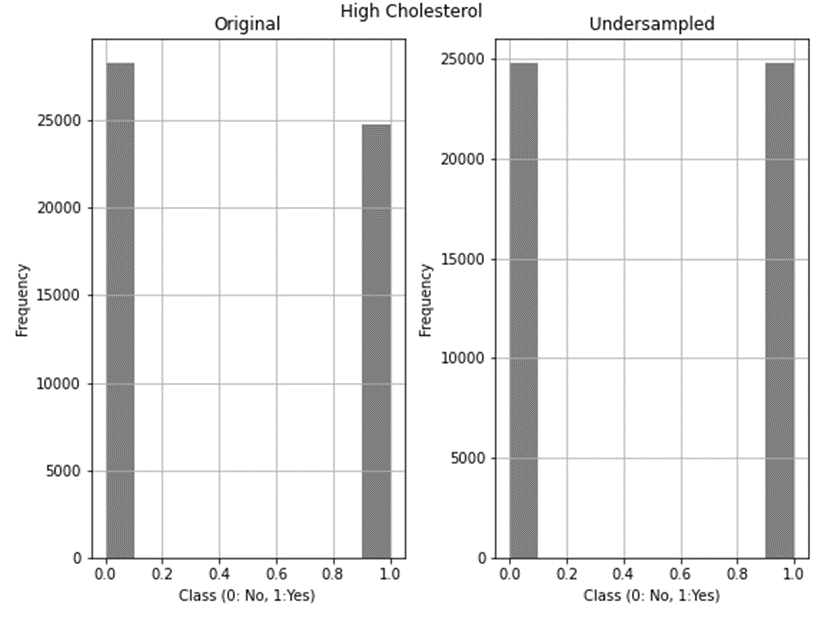


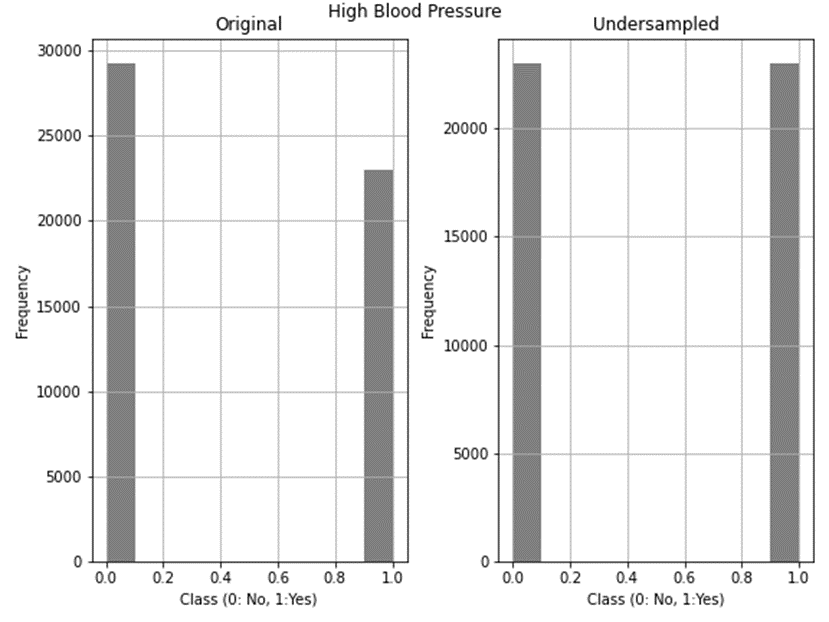


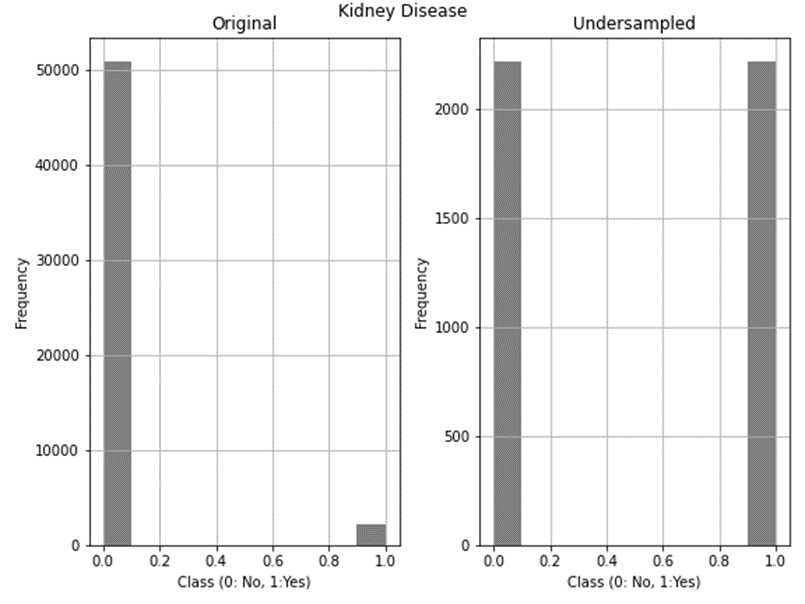


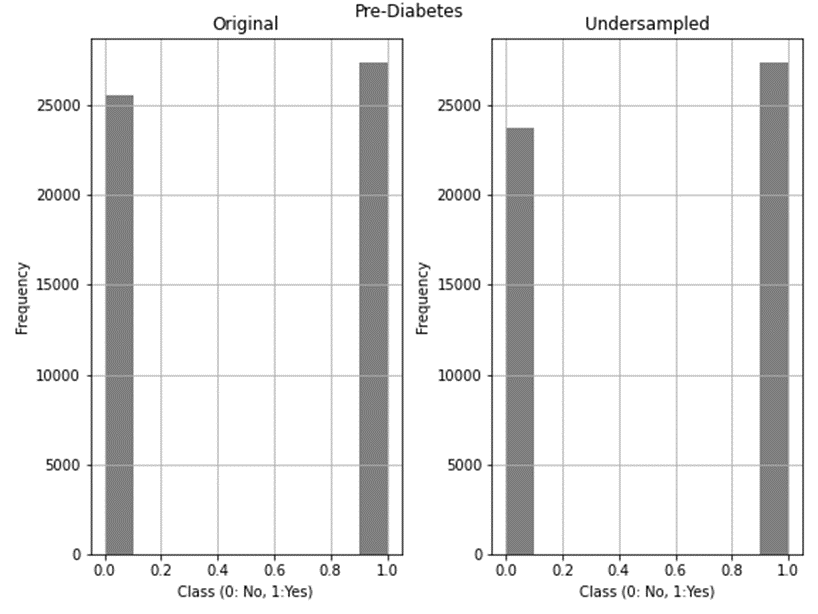


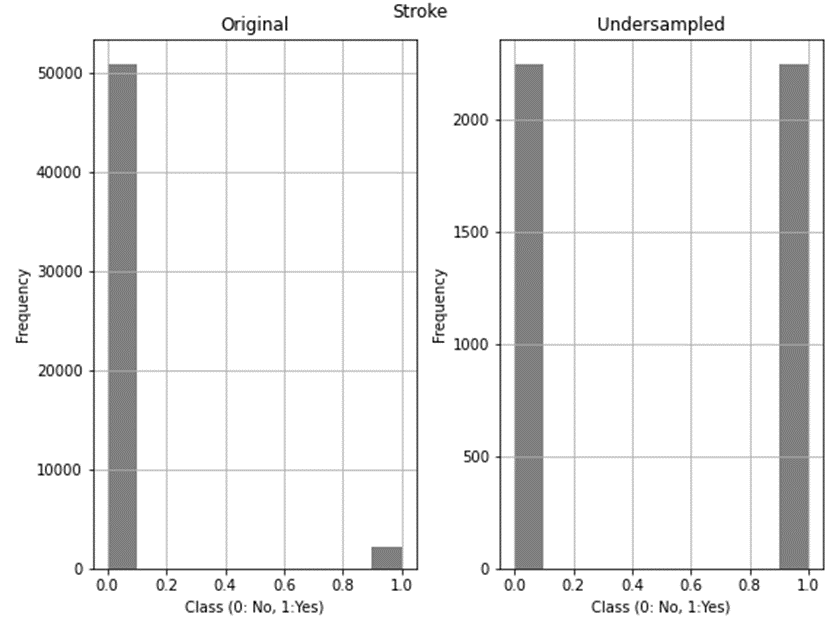


**Supplementary Figure S1.** Comparison of target variable distribution between original and undersampled data


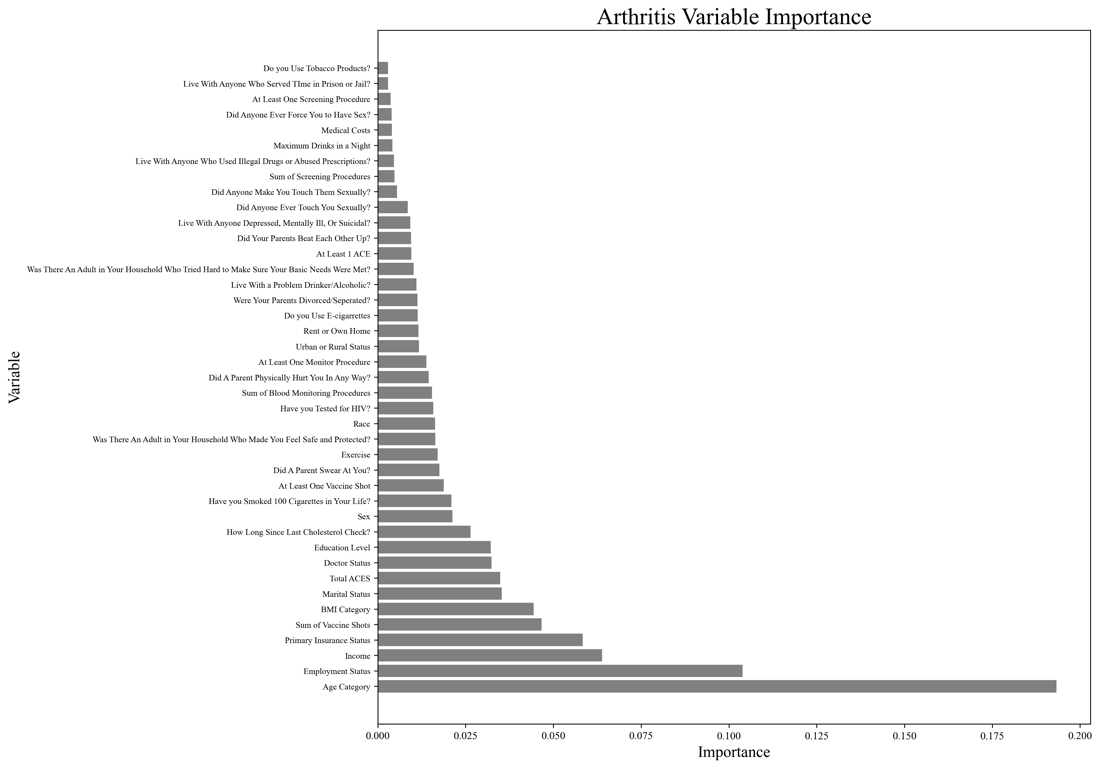


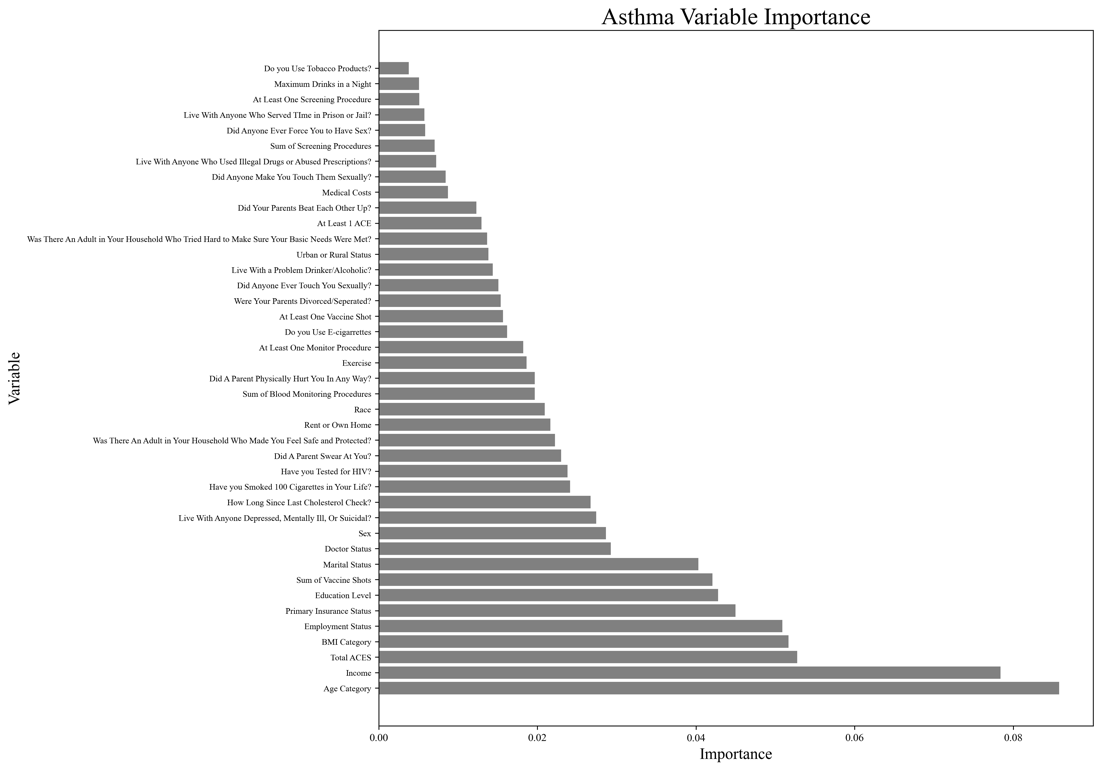


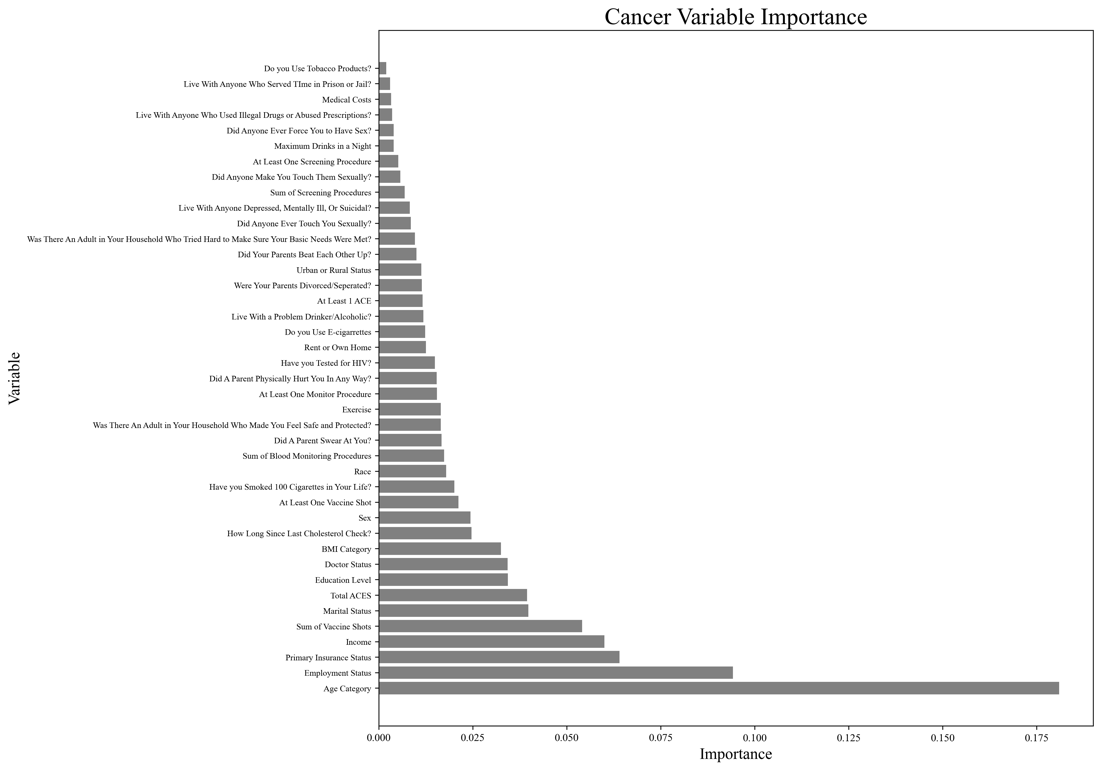


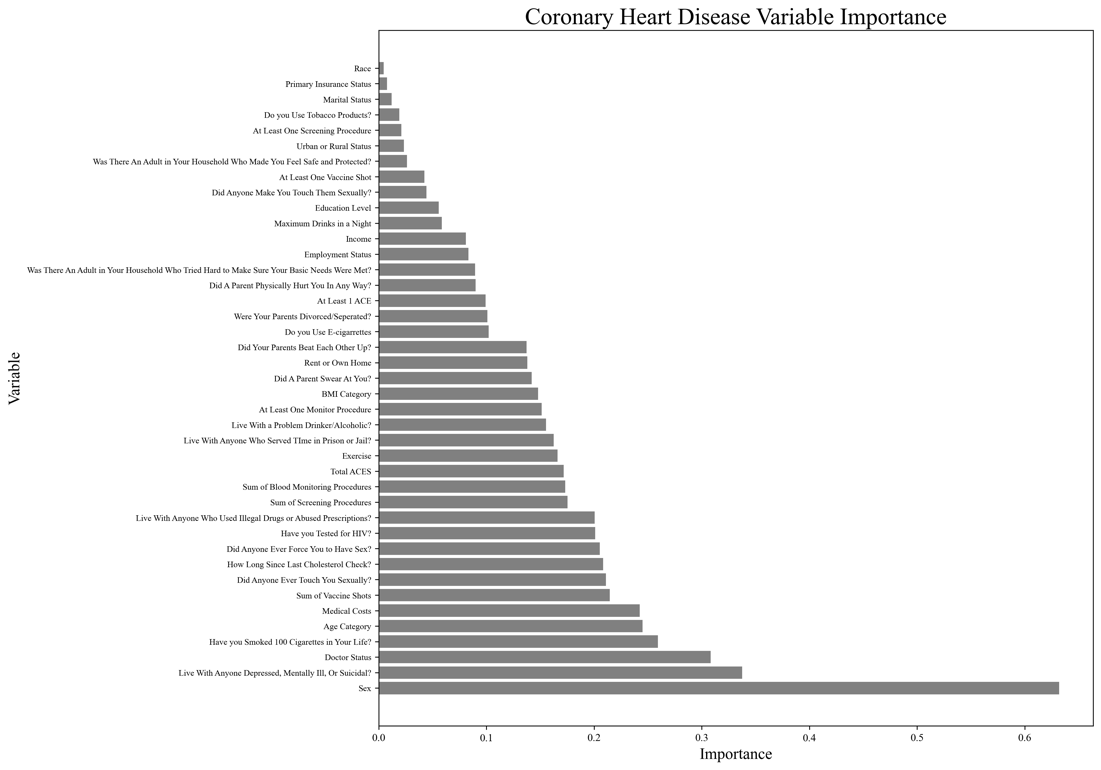

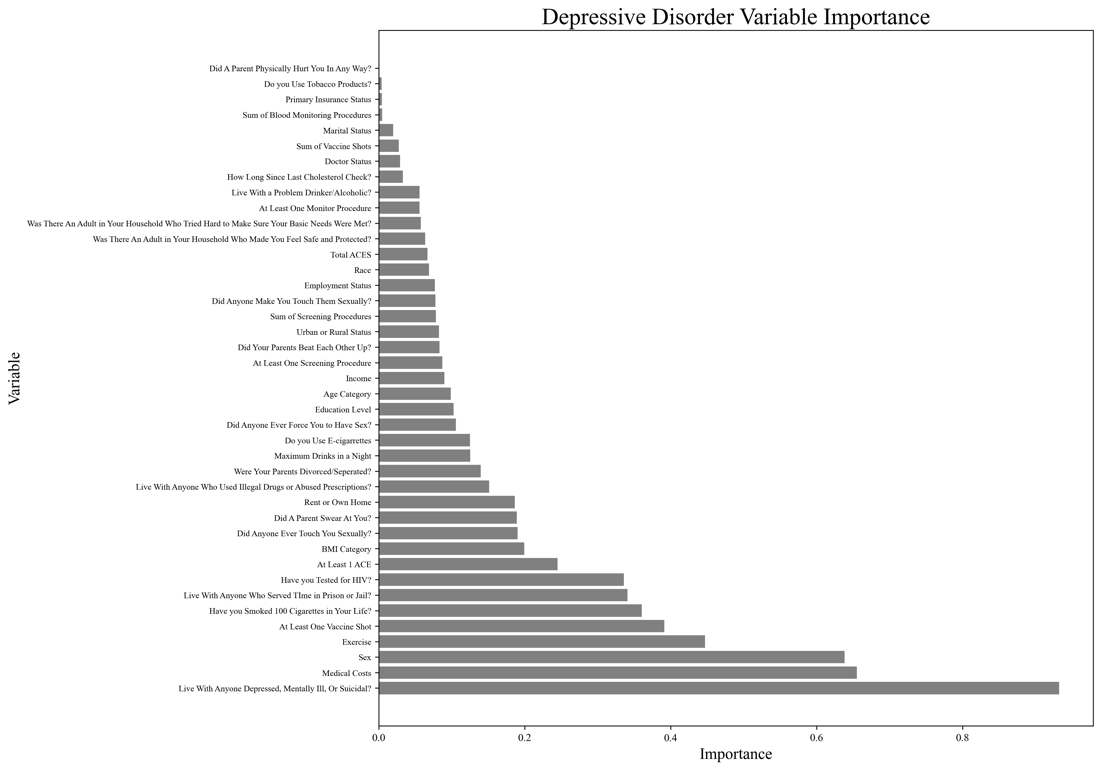


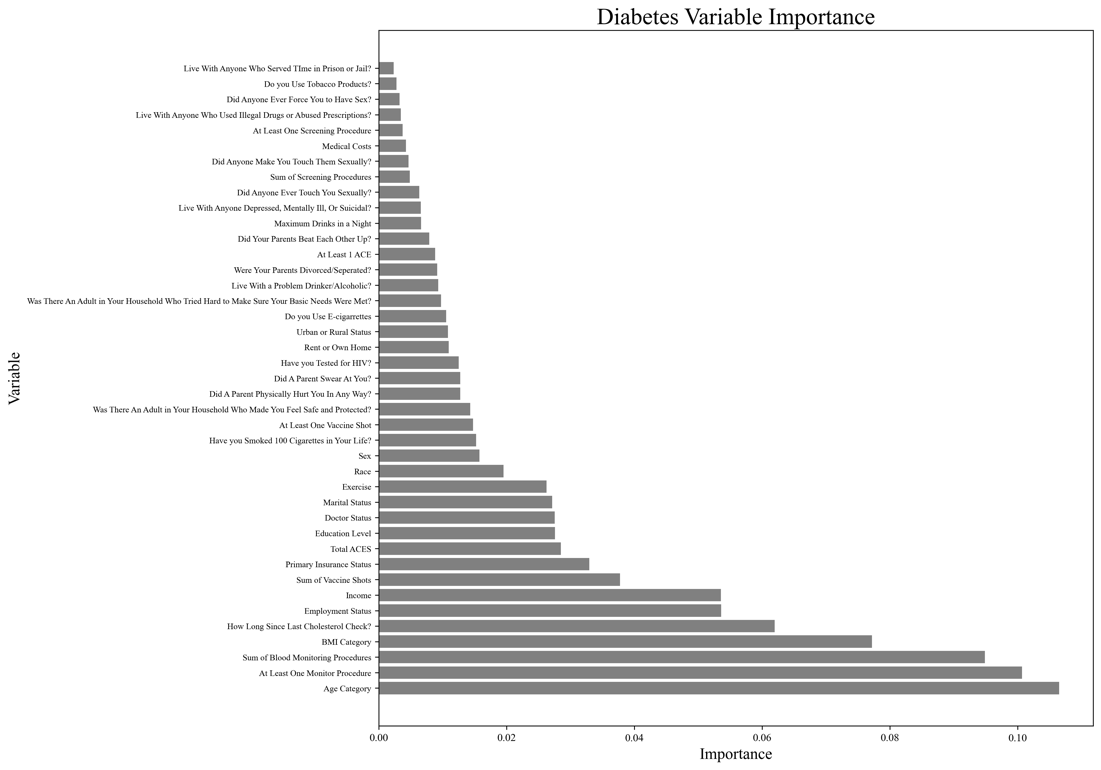


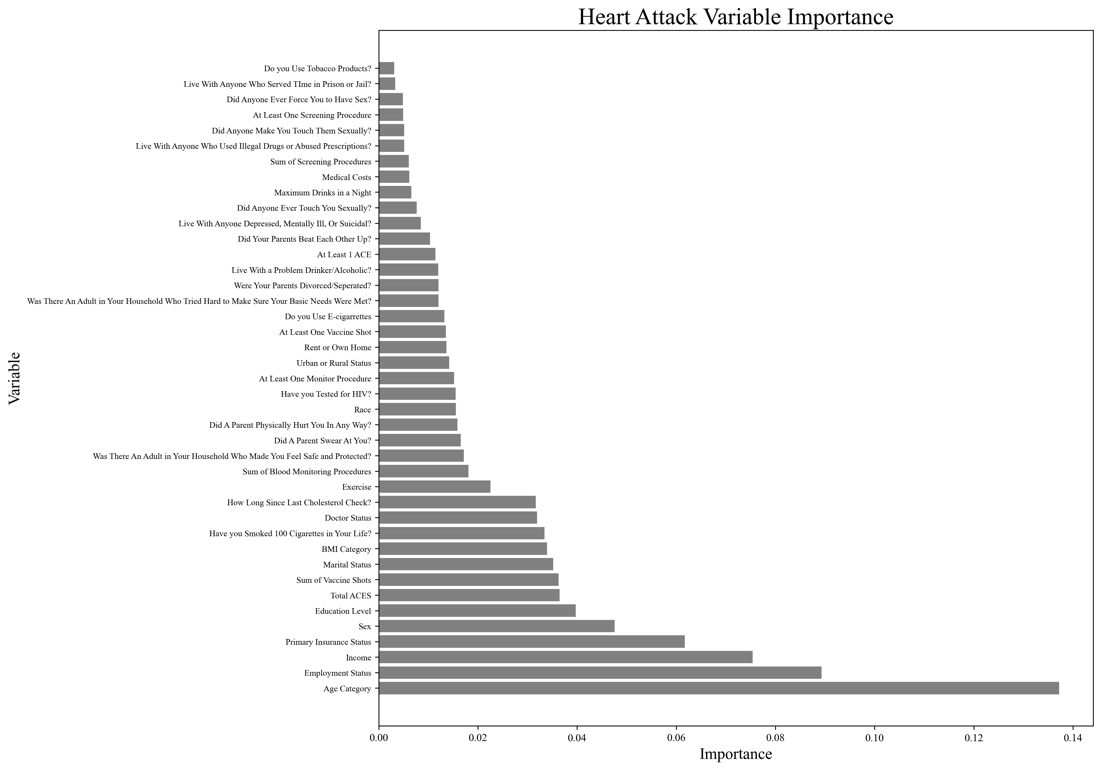


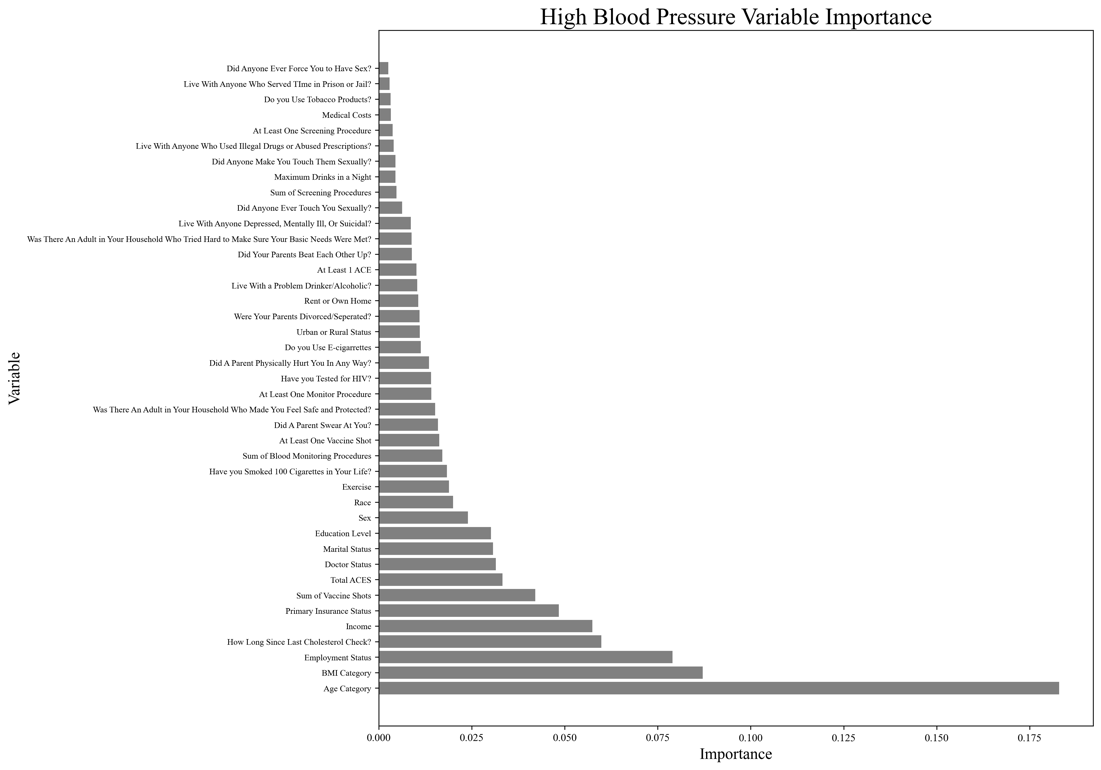


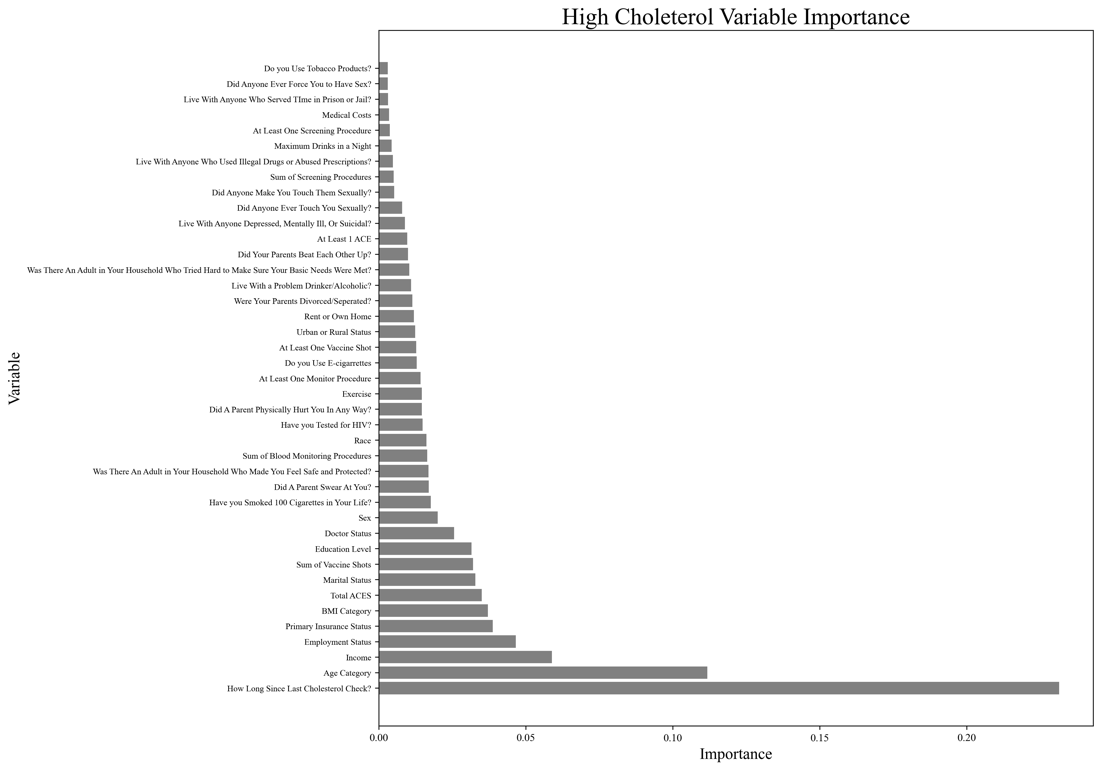


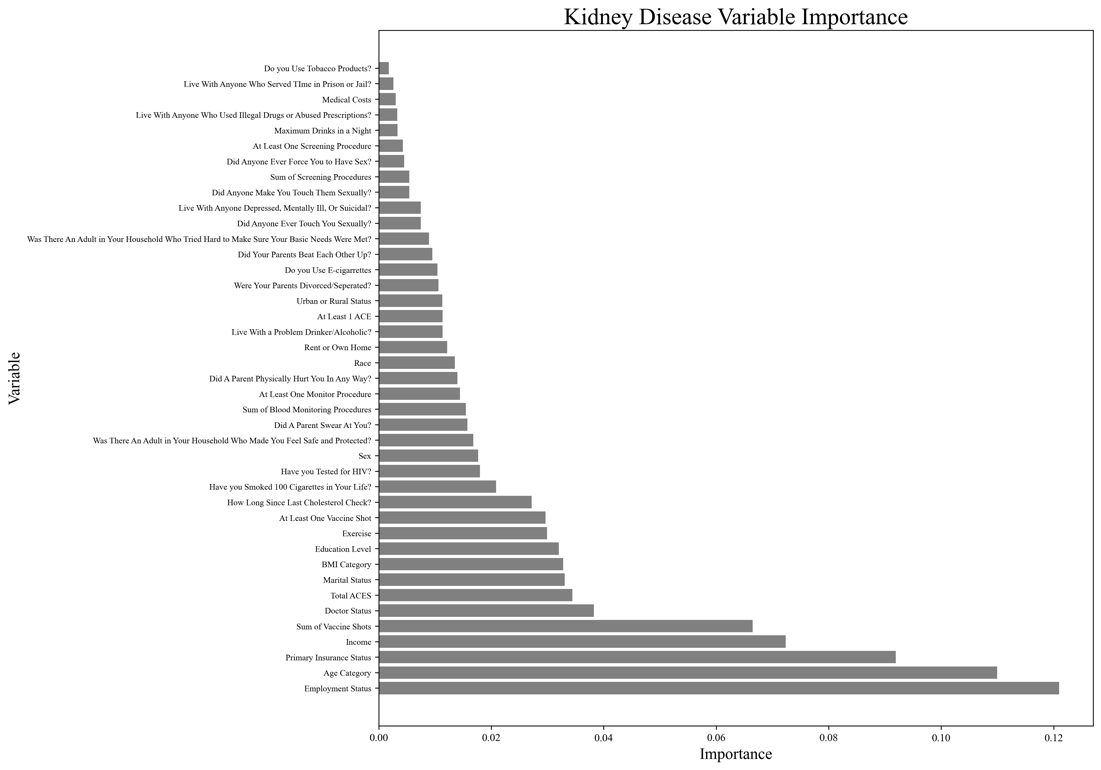


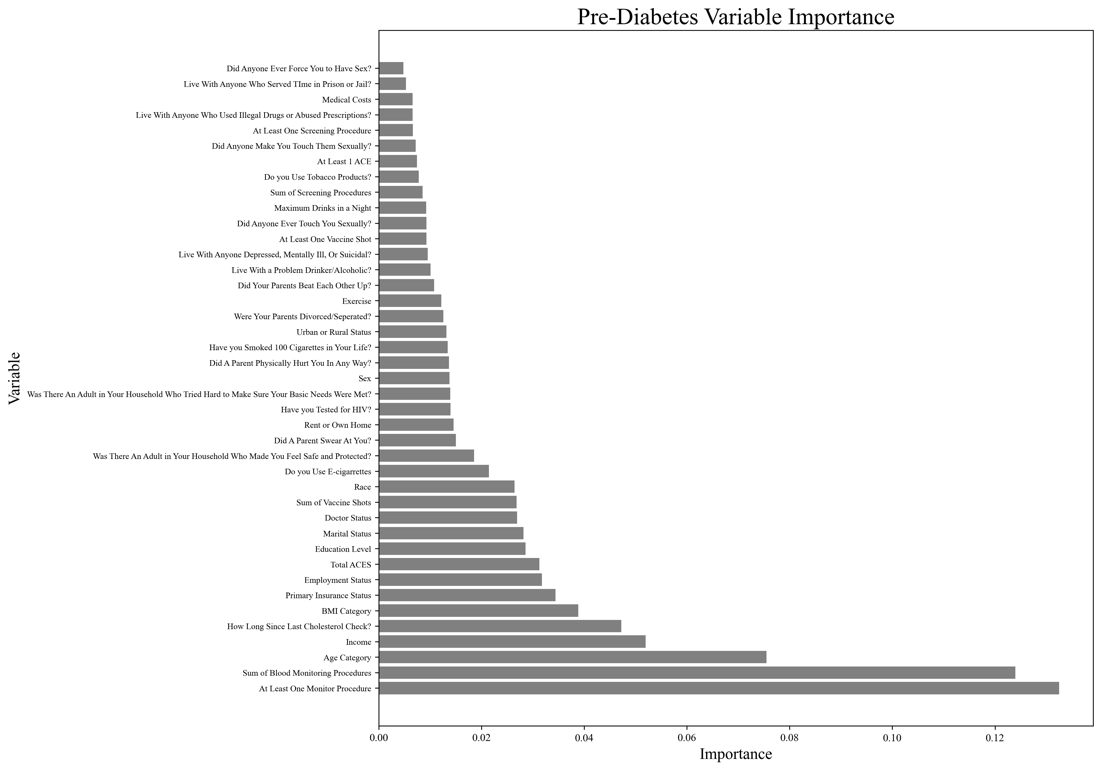


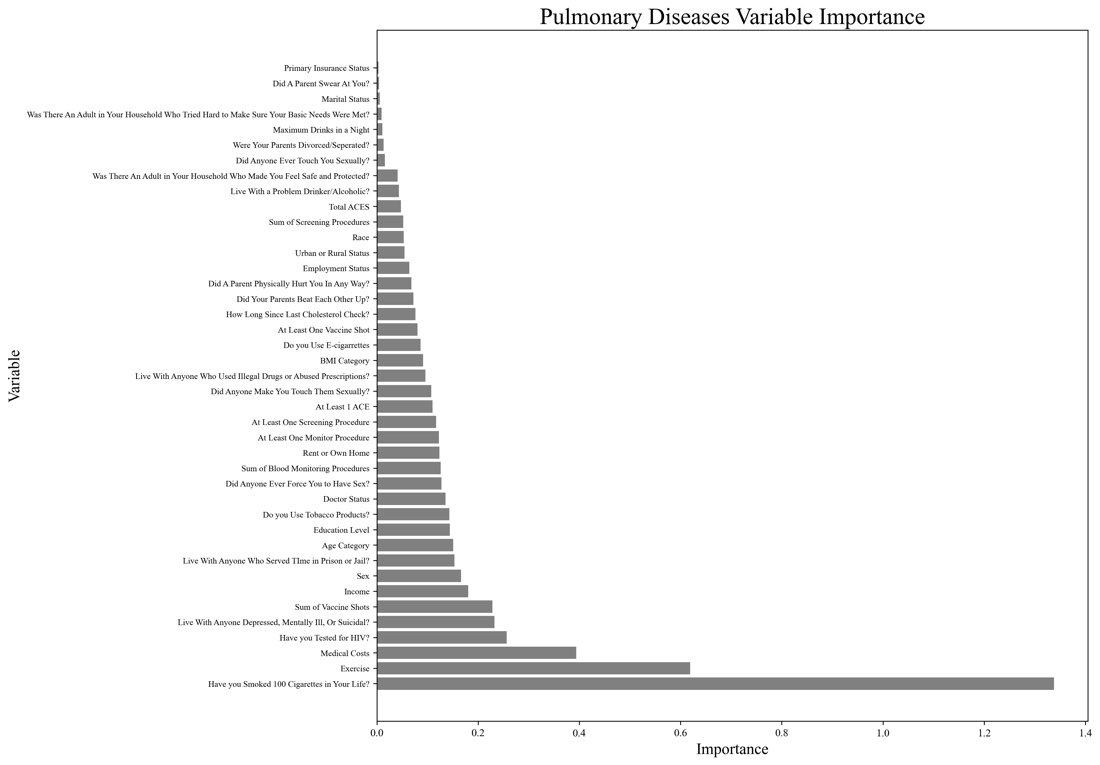


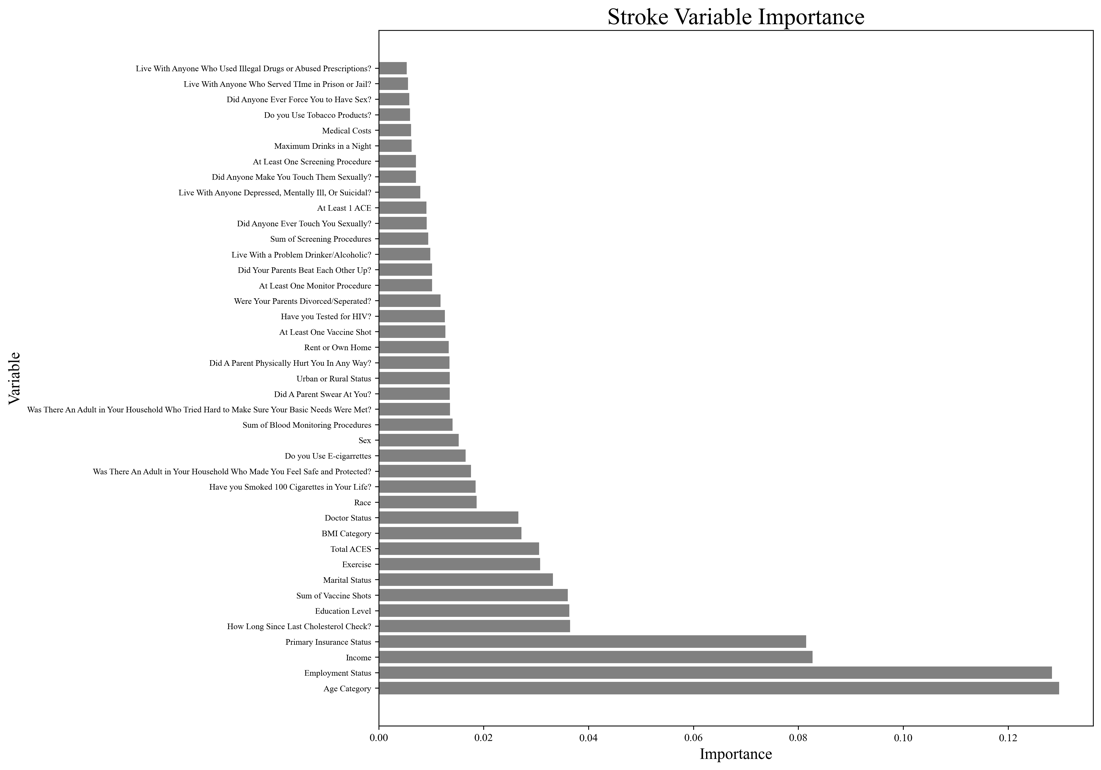


**Supplementary Figure S2.** Feature importance of each model
